# Anesthesia-Sepsis-Associated Alterations in Liver Gene Expression Profiles and Mitochondrial Oxidative Phosphorylation Complexes

**DOI:** 10.3389/fmed.2020.581082

**Published:** 2020-12-18

**Authors:** Hari Prasad Osuru, Umadevi Paila, Keita Ikeda, Zhiyi Zuo, Robert H. Thiele

**Affiliations:** ^1^Department of Anesthesiology, University of Virginia School of Medicine, Charlottesville, VA, United States; ^2^Center for Public Health Genomics, University of Virginia School of Medicine, Charlottesville, VA, United States

**Keywords:** cecal ligation and puncture (CLP), sepsis, subcellular energetics, OXPHOS, isoflurane, propofol

## Abstract

**Background:** Hepatic dysfunction plays a major role in adverse outcomes in sepsis. Volatile anesthetic agents may protect against organ dysfunction in the setting of critical illness and infection. The goal of this study was to study the impact of Sepsis-inflammation on hepatic subcellular energetics in animals anesthetized with both Propofol (intravenous anesthetic agent and GABA agonist) and Isoflurane (volatile anesthetic i.e., VAA).

**Methods:** Sprague-Dawley rats were anesthetized with Propofol or isoflurane. Rats in each group were randomized to celiotomy and closure (control) or cecal ligation and puncture “CLP” (Sepsis-inflammation) for 8 h.

**Results:** Inflammation led to upregulation in hepatic hypoxia-inducible factor-1 in both groups. Rats anesthetized with isoflurane also exhibited increases in bcl-2, inducible nitric oxide synthase, and heme oxygenase-1(HO-1) during inflammation, whereas rats anesthetized with Propofol did not. In rats anesthetized with isoflurane, decreased mRNA, protein (Complex II, IV, V), and activity levels (Complex II/III,IV,V) were identified for all components of the electron transport chain, leading to a decrease in mitochondrial ATP. In contrast, in rats anesthetized with Propofol, these changes were not identified after exposure to inflammation. RNA-Seq and real-time quantitative PCR (qPCR) expression analysis identified a substantial difference between groups (isoflurane vs. Propofol) in mitogen-activated protein kinase (MAPK) related gene expression following exposure to Sepsis-inflammation.

**Conclusions:** Compared to rats anesthetized with Propofol, those anesthetized with isoflurane exhibit more oxidative stress, decreased oxidative phosphorylation protein expression, and electron transport chain activity and increased expression of organ-protective proteins.

## Introduction

Volatile anesthetic agents (VAAs) have been shown to attenuate myocardial (1) and neurologic (2) injury after ischemic events in both animal and human studies, and small clinical trials have suggested a potential mortality benefit associated with the use of VAAs in patients undergoing cardiac surgery (who are at risk of both myocardial and cerebral ischemia) (3). The beneficial effect of VAAs administered prior to an insult is called anesthetic pre-conditioning (APC) and is mechanistically similar to but distinct from ischemic preconditioning (IPC) in which the insult is preceded by a brief period of ischemia and reperfusion, which then leads to protection (4).

Sepsis, which has a mortality rate of ~18–32%, is a significant global health problem, causing 721,000–2.1 million deaths/year, worldwide (5–8). While not traditionally used in the intensive care unit (ICU) environment, there is some evidence that VAAs may counteract the deleterious effects of sepsis on hemodynamics (9, 10), which may lead to improved mortality in animal models when compared to more traditional intravenous anesthetic agents- example, gamma-aminobutyric acid agonists (GABA agonists) (11, 12). The recent development of the AnaConDa™ (Sedana Medical, Uppsala, Sweden) and Mirus™ (Pall Medical, Dreieich, Germany) systems has made the use of VAAs in the ICU clinically practical, offering clinicians a novel strategy for ICU sedation which may also confer some degree of organ protection (13). In fact, a recent pilot study comparing VAAs to intravenous midazolam (GABA agonists) in humans with acute respiratory distress syndrome (ARDS) found reduced endothelial injury and inflammation as well as improved oxygenation in the VAA group (14).

The organ-protective mechanisms of APC are not fully understood but both APC and IPC appear to share activity on G-protein coupled receptors, protein kinase C, Mitogen-activated protein kinase (MAP kinases), Extracellular signal-regulated kinase (ERK), AKT serine/threonine-protein kinase (AKT kinase), hypoxia-inducible factor-1 (HIF-1), adenosine triphosphate (ATP)-sensitive potassium (K^+^) channels, and both free radical and nitric oxide (NO) production (15–17). All of these pathways intersect at the level of the mitochondria, which play a central role in the metabolic activity of eukaryotes (including during sepsis), through their production of ATP via the electron transport chain (ETC) as well as the production of reactive species as a byproduct of this process (18).

Makaryus et al. recently utilized proton magnetic resonance spectroscopy (pMRS) to better understand the differences of VAAs and GABA agonists on the metabolomic profile of the brain, finding that VAAs favored an anaerobic state (19). This data is consistent with prior work suggesting that VAAs inhibit Complex I of the ETC, leading to anaerobic metabolism and lactate production (19, 20). Propofol, by contrast, does not inhibit Complex I (21), but likely inhibits Complex II (22). The metabolic effects of anesthetics under septic conditions are not clearly understood. This knowledge may be useful to define the mechanisms of anesthetics-induced organ protection in critically ill patients. The primary purpose of this study was to compare the impact of inflammation on subcellular metabolism in the liver (one of the first organs affected by intra-abdominal infection) of animals exposed to VAAs (isoflurane) and GABA agonists (Propofol). In addition, we also evaluated oxidative stress and RNA-Seq profiles of Liver exposed to VAAs (isoflurane) and GABA agonists (Propofol).

## Materials and Methods

This study was approved by the animal care and use committee (IACUC) at the University of Virginia (protocol 4140) and conforms with the *Guide for the Care and Use of Laboratory Animals, 8th Edition* published by the National Institutes of Health (revised 2011). We have included additional methodological details in the Supplemental Digital Content-Methodology.

### General Preparation

Pre-surgical preparation, anesthesia, surgery (CLP) procedure monitoring and sample collection was performed as we previously described (23), 10-week-old (average weight 341 g) 48 male Sprague-Dawley rats (Envigo Corporation, Huntingdon, Cambridgeshire, UK) were induced with 5% isoflurane in 100% O_2_ for 12 min (isoflurane group) or 100–10 mg/kg intraperitoneally injected ketamine-xylazine (Propofol group). Rats were intubated orotracheally using a 16 ga. intravenous (IV) catheter, then mechanically ventilated (initial settings 1 Hz, 3.2 mL tidal volume). End tidal CO_2_ (ETCO_2_) was kept between 35 and 45 mm Hg. The right femoral artery and a tail vein or the right femoral vein were exposed and cannulated with 24 ga. IV catheters. Anesthesia was maintained with isoflurane (starting dose 1.5%, inspired) in 100% O_2_ or Propofol (average infusion rate 367 μg/kg/min, also 100% O_2_) and anesthetic depth was confirmed by toe pinching every 30 min. Temperature was measured and controlled to a target of 37°C with a rectal probe and a heating pad to a target of 37°C. A pulse oximeter was placed on the left lower extremity foot pad to measure arterial oxygen saturation (SpO_2_).

### Surgical Procedure(s) and Sample Harvesting

After obtaining vascular access, 2 mL of arterial blood was withdrawn and replaced with normal saline (NS, 37°C). Two hundred microliter of each sample was used for arterial blood gas (ABG) analysis (Chem8^+^ and CG4^+^, Abbot i-Stat, Lake Bluff, IL) and 1.8 mL was injected into a serum collection tube and stored at room temperature for 45 min, after which it was centrifuged at 4°C. Serum was then separated and stored at −80°C.

Abdominal hair was shaved using an electric razor and cleaned with three 70% isopropyl alcohol swabs, followed by scrubbing with a 2% chlorhexidine gluconate and 70% isopropyl alcohol prep stick. A sterile drape was applied and a 2–3 cm midline celiotomy was performed using sterile technique. For isoflurane (24 total) and Propofol-based (24 total) anesthetics, animals were divided into controls (C) and cecal ligation and puncture (CLP) groups in alternating fashion. In the control (sham surgery) group, the cecum was removed from the abdomen and immediately returned to the abdominal cavity. In the inflammation group, 90% of the cecum (by volume) was ligated with a size 0 silk suture, 10 holes were placed using a 16-gauge needle (mesenteric border and anti-mesenteric borders), and patency was confirmed through extrusion of extruding fecal contents. The celiotomy was closed with 4–0 running silk sutures through the muscular layer, followed by skin clips. The animal was turned prone, a halogen light source trans-illuminated the skull, and near infrared spectroscopy (NIRS) recordings were initiated. Hypotension (mean arterial pressure < 80 mm Hg) was first treated with warm normal saline (NS) (up to 3.3 mL/kg/hr) given intravenously. If ineffective, the dose of anesthetic was reduced (and depth of anesthesia confirmed) when possible.

General anesthesia was maintained for 8 h post-celiotomy, after which 2 mL of arterial blood was withdrawn. Bilateral burr holes were placed and the supratentorial brain was ejected onto a liquid N_2_-cooled copper plate by applying a high-pressure air hose to one of the burr holes (freeze blow technique, see brain data published previously) (23). Immediately after death of the animal, the abdominal closure was re-opened, a piece of liver (~25% by volume) was removed and placed immediately into liquid nitrogen.

For biochemical analysis, all tissues were removed from −80°C storage and finely ground in liquid N_2_ using a mortar and pestle. Ten to fifty milligram of tissue powder (per sample) were used to measure protein expression, markers of oxidative stress, ATP levels, and other molecular studies.

### Enzyme-Linked Immunosorbent Assays (ELISA)

As we described previously (23), Arterial blood was placed in BD Vacutainer tubes (Becton Dickson, Franklin Lakes, NJ) after collection, hand-mixed, and stored at room temperature for 45 min, after which it was centrifuged (2,000 g) for 20 min at 4°C. The serum was removed with a micropipetter and stored at −80°C for later analysis. Interleukin (IL)-6 (RAB0311, Sigma, St. Louis, MO), Interleukin (IL)-1β (RAB0277, Sigma), and tumor necrosis factor (TNF)-α (ab100785, Abcam, Cambridge, UK) were measured in accordance with the manufacturer's instructions. For all assays, standards and unknowns were both measured in duplicate.

### Liver ATP Levels

Liver mitochondrial ATP levels were measured using Bioluminescent Assay kit (MAK135, Sigma) according to the manufacturer's protocol. Briefly, a working reagent was added to the extracted mitochondria (Cayman Chemicals #701010, Ann Arbor, MI) to inducing lysis and releasing ATP. The addition of luciferase and D-Luciferin to the lysed product allowed for the measurement luminescent intensity (ATP) of the sample using Synergy™ Multi-Mode Microplate Reader (BioTek, USA). All the samples were read in duplicate and purified ATP (Abcam, ab 83355) used as a standard.

### Immunoblotting

Rat liver tissue (50 mg), were used either for nuclear protein extraction or for whole cell lysate preparation, depending on the protein to be analyzed. As we described previously (23), Nuclear proteins were extracted using a commercially available nuclear extraction kit (Abcam ab113474, Cambridge, MA) in accordance with the manufacturer's protocol, with the exception of adding 100 uM of the prolyl hydroxylase stabilizer CoCl_2_ to the extraction buffer (RIPA) to prevent hypoxia-inducible factor-1α (HIF-1α) degradation in the presence of atmospheric oxygen.

For whole cell lysate extraction, 250 uL of ice-cold lysis buffer (RIPA) containing Halt™ protease and phosphatase inhibitor cocktail was added to the 50 mg of liver tissue powder, which was sonicated on ice (3 cycles of 10 pulses, power 5, Fisher Scientific Sonic Dismembrator Model F60, Pittsburgh, PA). Lysates were then centrifuged (13,000 rpm) for 20 min at 4°C and supernatant was collected.

Protein concentrations were measured using a Bicinchoninic Acid (BCA) kit (Thermo Scientific). Ten to Thirty microgram of whole cell lysate (50 μg for HIF-1α) was heat-denatured at 95C, loaded onto 4–20% Tris-Glycine polyacrylamide gradient gels (Bio-Rad, Hercules, CA), and electrophoresed. Proteins were transferred to PVDF membrane (Millipore, Darmstadt, Germany), blocked for 1 h at room temperature in SuperBlock phosphate-buffered saline (PBS) Blocking Buffer (Thermo-Fisher # 37515) and incubated with primary antibodies at 4°C overnight. Specific antibodies utilized included: anti-HIF-1α (1:2,000, rabbit-mAb # 14179, Cell Signaling [Danvers, MA]; note: for HIF-1α, overnight incubation with primary antibodies was repeated after washing three times with PBST), anti-heme-oxygenase-1 (HO-1, 1:3,000, mouse- mAb # NBP1-97507, NovusBio [Littleton, CO]), or anti-B-cell lymphoma 2 (bcl2, 1:5,000, mouse- mAb # NB100-78543, NovusBio), anti-inducible nitric oxide synthase (iNOS, 1:1500, rabbit-mAb # 13120, Cell signaling). For loading controls, anti-β-tubulin (1:2,000, mouse-mAb # NB 120-7792, NovusBio) and anti-Lamin B1 (1:2,000, rabbit-mAb # 13435, Cell Signaling) were used. After primary antibody incubation, the PVDF membranes were washed three times in PBST for 10 min. PVDF membranes were then incubated with horseradish peroxidase–conjugated secondary antibodies (1:1,000–15,000, depending on the primary antibody used, Santa Cruz Biotechnology [Dallas, TX] and Cell Signaling], see Supplemental Digital Content-Methodology section for more details) at room temperature for 1 h. Immunoreactivity was detected using Super Signal West Femto (Thermo Scientific), and enhanced chemiluminescence substrate. Images were captured using GBOX (Chemi XR5; Syngene), and blots were analyzed densitometrically using the computerized image analysis software (Gene Tools from Syngene). Protein bands were normalized to loading controls (β-tubulin or Lamin B1) and expression levels were presented in percentage.

### Measures of Oxidative Stress: Protein Carbonyl Assay

Protein carbonyls levels are widely used to measure protein oxidation. As previously described (23). In brief, 50 mg of Liver tissue powder was suspended in ice cold PBS + 0.5 mM EDTA buffer containing a protease and phosphatase inhibitor cocktail (Halt™), followed by sonication and centrifugation at 4°C. To remove nucleic acids from the supernatant, 10% streptozocin was added and the supernatant was incubated at room temperature. After re-centrifugation, 5 uL of sample were utilized for the measurement of protein concentration (BCA assay), and the remainder was used for the Protein Carbonyl Content Assay Kit (MAK094, Sigma-Aldrich, St. Louis, MO) in accordance with the manufacturer's instructions. 2,4-dinitrophenylhydrazine (DNPH) was added to each sample, followed by vortexing and incubation at room temperature. TCA was added followed by repeat vortexing, incubation on ice, centrifugation at 4°C, and removal of supernatant. Acetone was added to the pellet which was then sonicated and centrifuged. Guanidine solution was added and sonication repeated, after which absorbance was measured at 375 nm (A_375_), allowing for the calculation of carbonyl content.

### S-Nitrosylation Assay

As previously described (23), liver ground tissue powder was lysed in HENS buffer containing 100 mM HEPES (pH 7.8), 1 mM EDTA, 0.1 mM Neocuproine, and 1% SDS, followed by sonication and centrifugation at 4°C. Supernatant was collected and the protein concentrations were determined using a BCA kit (Thermo Scientific). Two microliter of 1 M methyl methanethiosulfonate (MMTS) was added to each sample, which was then vortexed and incubated at room temperature. Pre-chilled acetone was added to the samples and incubated at −20°C, in order to precipitate the protein. The samples were centrifuged, the pellet was air dried, and then re-suspended in HENS buffer. Sodium ascorbate and iodo-TMT labeling reagent were added, vortexed, and incubated in a cold room overnight. Pre-chilled acetone was added to the samples which were incubated at −20°C, re-centrifuged at 10,000 rpm for 15 min, then the pellet was air dried and re-suspended in HENS buffer. 12.5 ul of 4X reducing Laemmli sample buffer was added to the iodo-TMT-labeled samples, and heated at 99C for 5 min. Twenty microliter of each sample was loaded onto 4–20% TGX gels and electrophoresed. Proteins were then transferred to PVDF membrane, blocked for 1 h at room temperature in SuperBlock Blocking Buffer (Thermo-Fisher # 37515), then incubated at 4°C overnight with anti-TMT antibody (1:2,000, Thermo-Fisher # 90075). Membranes were then washed three times in TBST for 10 min then incubated with anti-mouse IgG-HRP conjugated (1:15,000) secondary antibody at room temperature for 1 h. Immunoreactivity was detected using enhanced chemiluminescence substrate (Super Signal West Femto; Thermo Scientific). Images were captured using GBOX (Chemi XR5; Syngene), and gels were analyzed densitometrically using the computerized image analysis software (Gene Tools from Syngene). Loading controls (total protein) were stained with Ponceau S solution and S-nitrosylation was normalized to Ponceau S stain of total protein.

### Serum Nitric Oxide Levels

Total serum nitric oxide levels were estimated using a colorimetric assay kit that measures total nitrate, nitrite levels (Nitric Oxide Assay Kit# EMSNO, Invitrogen). All the serum samples were diluted (1:2) with assay dilution buffer and filtered through a 10,000 MWCO spin columns (ab93349, Abcam) and 50 ul was used for assay. Nitrate, Nitrite concentrations were determined by spectrophotometric analysis at 540 nm (Synergy™ Multi-Mode Microplate Reader (BioTek, USA) and the total serum nitric oxide levels were calculated by using standard curve and according to the manufacturer's protocol. All the samples were read in duplicate and NO products were expressed as μmoles.

### Mitochondrial Isolation and Complex I-V Activity Assay

#### Mitochondrial Isolation

Rat liver mitochondria was isolated using the MitoCheck® Mitochondrial Isolation Kit (Cayman Chemicals #701010, Ann Arbor, MI) by following the manufacturer's instructions. Briefly, 1.2 gm of Liver tissue was rinsed in 2 mL of cold mitochondrial isolation buffer (MIB) to remove blood. Tissue pellet was suspended in 2 mL of mitochondrial homogenization buffer (MHB), homogenized with Dunce Tissue Homogenizer for 10Sec X 3 times on ice and centrifuged at 1,000 rpm for 10 min at 4°C. Supernatant was collected to a new pre-chilled 2 ml tube and filled with remaining MHB buffer, centrifuged at 10,000 rpm for 10 min at 4°C. The pellet was re-suspended in 500 μL of cold MIB buffer and the protein concentration was measured using BCA kit (Thermo Scientifics) with bovine serum albumin as a standard.

#### Mitochondrial Complex Activity Assays

For Complex I-V activity assays 10 μg of control or test liver mitochondrial protein extract was diluted in 20 ul of complex assay buffer. All samples were measured in duplicate using Synergy™ Multi-Mode Microplate Reader (BioTek, USA). All the MitoCheck Complex Activity Assay Kits were purchased from Cayman Chemicals, MI and all the activity assays was performed according to the manufacturer's protocols.

The activity of complex I (NADH oxidase/co-enzyme Q reductase, Kit #700930) was analyzed by measuring the decrease in NADH oxidation, which is reflected by a decreased in absorbance at 340 nm in the presence of 100 mM potassium cyanide (KCN) to prevent the oxidation of co-enzyme Q reductase. The activity of complex II/III (Kit #700950) is determined by measuring the complex III-dependent reduction of cytochrome c (Cyt c) in mitochondrial sample, which is reflected by increased absorbance at 550 nm. Complex II/III activity were performed in the presence of 1 μM Rotenone and 2 mM KCN, to prevent backflow of electrons through complex I and the reduction of cytochrome c by complex IV. Mitochondrial Complex IV (cytochrome *c* oxidase, Kit# 700990) was determined by measuring the rate of oxidation of cytochrome c, due to oxidization of the reduced cytochrome c, which is reflected by a decrease in absorbance at 550 nm. Mitochondrial Complex V (F_1_F_0_ ATP Synthase, Kit# 701000) activity was analyzed by the rate of NADH oxidation, which can be measured by a change in absorption at 340 nm. Complex I also oxidize NADH, to prevent Complex I interference we performed Complex V assay in the presence of 1 μM Rotenone.

#### Liver Mitochondrial OXPHOS Levels

To evaluate the levels of total OXPHOS in liver, 25 μg of isolated, pooled mitochondria (see Supplementary Table 1) were resuspended in 5 X SDS-PAGE sample loading buffer, incubated at room temperature for 30 min. Proteins were separated on 4–20% PROTEAN® TGX™ Precast Protein Gels (Bio-Rad), and was analyzed by Western blotting. Blots were probed with a total OXPHOS Rodent WB Antibody Cocktail diluted 1:1,000 in washing buffer (ab110413; Abcam, Cambridge, UK) and selective for each of the 5 electron transport complexes (*I*–*V*). Ponceau S-staining and mitochondrial protein anti-HSP60 (1:5,000, rabbit-mAb #GTX110089, GeneTex) was used for loading control and for the normalization for the densitometric quantification.

#### Liver Mitochondrial DNA Levels

DNA was extracted from frozen Liver tissue (20 mg) using DNeasy Blood & Tissue kit (#69504, Qiagen). DNA quality and quantity were estimated using a Nanodrop spectrophotometer (Thermo Scientific). To estimate the amount of mtDNA relative to nDNA, we used two sets of primers encoding a mitochondrial gene (*Mt_Nd1* and *Mt_Nd6*) and a nuclear gene Tubulin (*Tuba1*) (24). iTaq universal SYBR® Green supermix (BioRad, USA) and 50 ng of DNA was used and amplification was conducted using CFX Connect Real-time qPCR system (Bio Rad, USA). The relative mtDNA content was calculated using ΔΔCT method (25). Accuracy of the target amplicon was checked with a melting curve, and each sample was read in triplicate.

#### RNA Isolation, Library Preparation, Sequencing, Quality Control, and RNA Expression Analysis

To better understand the interaction between sepsis-inflammation and anesthetic agents, we analyzed mRNA expression for the two groups of rats anesthetized with isoflurane or Propofol and contrasted CLP with controls/sham (no CLP) in both these groups. Differential expression analysis was performed with DESeq2 (26) and statistically significant differences were then screened by the authors with a focus on genes related to oxidative phosphorylation (OXPHOS), energy metabolism, cellular protection, and oxidative stress. The pathway enrichment was performed with clusterProfiler from the R package (27) and the heatmaps were generated with the ComplexHeatmap package in R (28).

Tissue pooling was performed as previously described (23) and is reported in Supplementary Table 1. In brief, we used a commercial kit for total RNA isolation (RNeasy plus mini kit, Qiagen) and NanoDrop (ThermoFisher Scientific) to estimate the RNA concentrations. Two hundred nanogram of each sample was sent to our Genome Analysis Technology Core for mRNA sequencing using Illumina Next-Generation. Technical details of sequencing, including total RNA quantification, library selection, quality control, and RNA expression analysis were previously reported (23).

To focus our results on key pathways known to be related to inflammation and subcellular energetics, the following screening search terms were employed: angiogenesis, cytochrome, oxidative, stress, hsp, electron, heat, atp, metabolic, apoptotic/apoptosis, mitochondria, complex, hypoxia, mapk, and NF-κB pathway. All processed data were uploaded to the gene expression omnibus (https://www.ncbi.nlm.nih.gov/geo/; accession # GSE138454).

#### Real-Time RT-PCR (Qpcr)

To estimate and validate the NGS-RNAseq- mRNA expression levels, we selected list of mitochondrial OXPHOS complex I-V genes that encode specific mitochondrial complex subunits, along with a housekeeping reference gene. Total RNA (2–5 μg) was reverse transcribed using iScript-Adv cDNA Synthesis Kit (BioRad, USA). The resulting cDNA (50–100 ng) was subjected to qPCR analysis in a final volume of 20 μl containing, iTaq universal SYBR® Green supermix (BioRad, USA) and gene specific primers (Supplemental Digital Content). Amplification was conducted using CFX Connect Real-time PCR system (BioRad, USA). Fold changes in expression were calculated using the ΔΔCT method (25), Each reaction was run in triplicate and alpha Tubulin was used as a normalization control.

#### Power Analysis and Statistical Comparisons

This study is a secondary analysis of a study designed to better understand the impact of sepsis on subcellular energetics and which was powered to detect the change in the oxidation state of cytochrome aa3 in the brain [see (23)]. All data were tested for normality using the D'Agostino-Pearson test. Normally distributed groups were compared using the *t*-test (parametric) or Mann-Whitney test (non-parametric) as appropriate. When paired data were available (e.g., interleukins), paired tests were utilized to account for interindividual variability. All calculations were performed in GraphPad Prism-8 (GraphPad Software, La Jolla, CA) and MATLAB (Mathworks, Natick, MA).

Unless otherwise noted, parametric grouped data are presented as mean and 95% confidence intervals, whereas non-parametric grouped data are presented as median and interquartile range.

## Results

**Figure 1:**
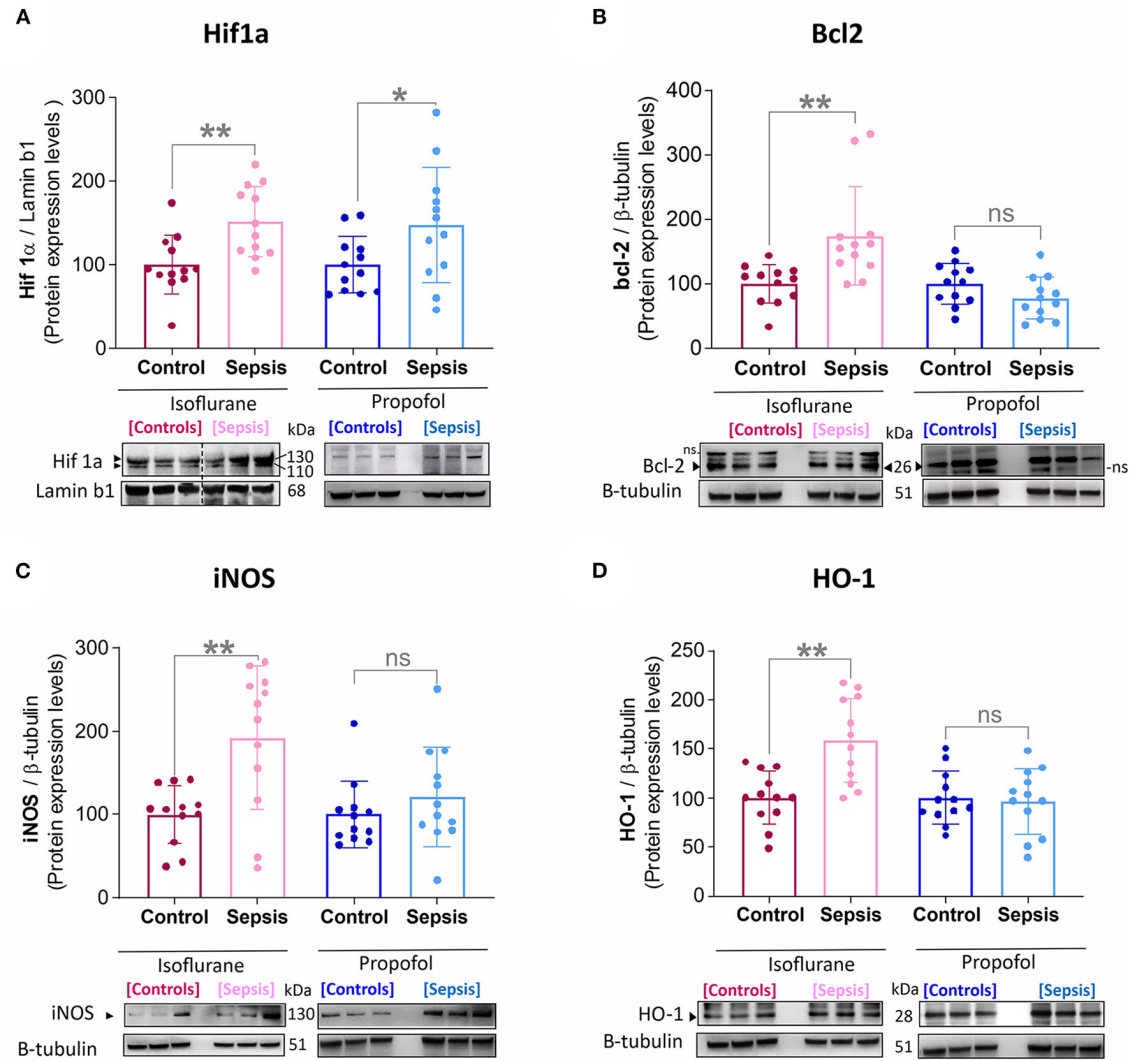
Effect of Sepsis with Isoflurane or Propofol anesthesia on Liver HIF1a, Bcl2, iNOS and HO-1, Protein expression levels. **(A)** HIF-1α protein was increased in both anesthetic groups by sepsis (Isoflurane +Sepsis, **p = 0.004 and Propofol + Sepsis, *p = 0.04). **(B)** Bcl-2 protein was increased in Isoflurane +Sepsis group (**p = 0.004) and no significant change in Propofol + Sepsis (*p = 0.104). **(C)** Inducible nitric oxide synthase [iNOS, (**p = 0.002)] and **(D)** Heme oxygenase-1 (HO-1, ***p = 0.0006) was increased in Isoflurane +Sepsis group. Whereas, in animals anesthetized with Propofol, three organ-protective proteins, Bcl-2, iNOS, and HO-1 expression was unchanged significantly. Data are shown in mean ± S.D, *n* = 12. *denotes a statistically significant (*P* ⩽ 0.05) difference between animals exposed to sepsis and controls. Statistical differences between results were evaluated using the *t*-test. “*ns*” (in graphs) represents not statistically significant. “*ns*” (in immunoblots) represents non-specific signal. “◂” represents specific signal.

**Figure 2:**
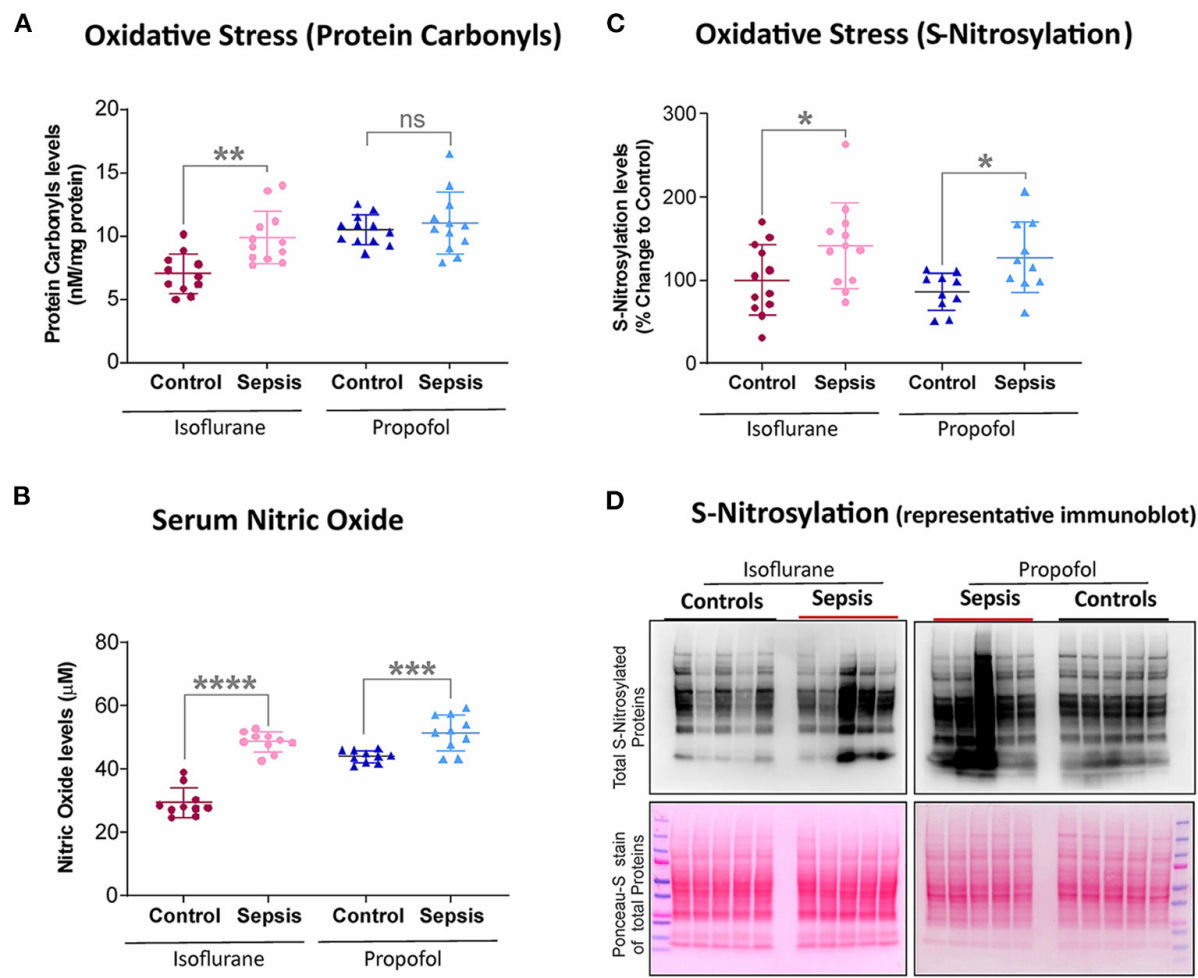
Effect of Sepsis with Isoflurane or Propofol anesthesia on Liver Oxidative stress markers. **(A)** Liver protein carbonyl content was increased in isoflurane sepsis (**p = 0.001) and unchanged (*p* = 0.511) in Propofol + Sepsis group. **(B)** Serum nitric oxide levels was significantly increased in both isoflurane sepsis (****p = 0.0001) and Propofol + Sepsis groups (***p = 0.0009). **(C)** S-nitrosylated protein levels was increased in Isoflurane +Sepsis group (*p = 0.043) and Propofol + Sepsis groups (*p = 0.014). **(D)** A representative immuno blots displaying total S- nitrosylated protein and total proteins (loading control) of Ponceau- S staining. *n* = 10–12 animals/group; Bars are mean ± S.D. “*ns*” (in graphs) represents not statistically significant.

**Figure 3:**
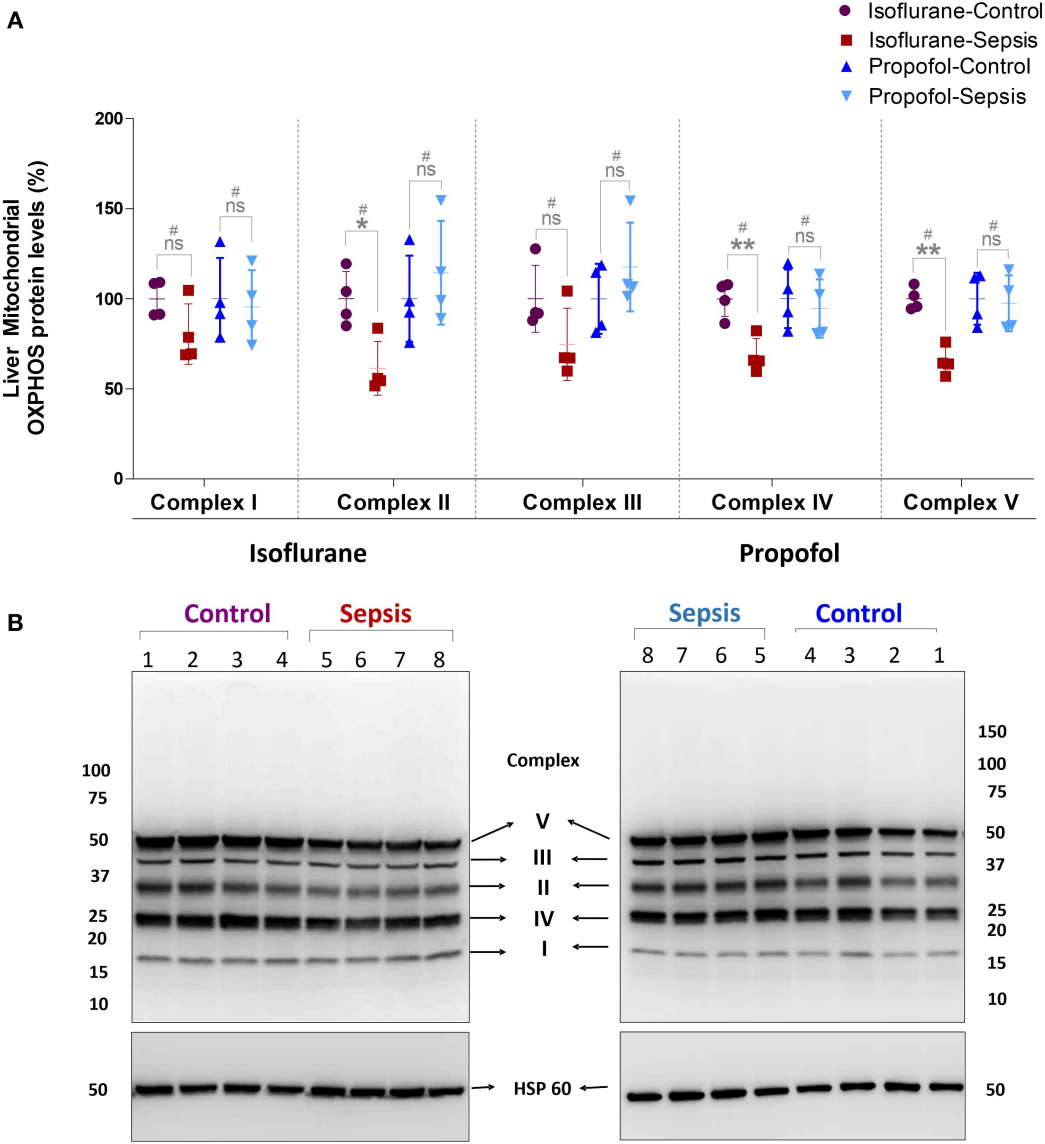
Effect of Sepsis with Isoflurane or Propofol anesthesia on Liver Mitochondrial Oxidative Phosphorylation (OXPHOS) Complex I-V, Protein expression levels. **(A)** Western blot analysis showing OXPHOS-Complexes I-V Protein levels, Complex-II (*p = 0.011), Complex-IV (**p = 0.003) and Complex-V (**p = 0.005) protein levels was significantly down regulated in isoflurane sepsis group and the complex protein expression levels are not significantly effected in Propofol Sepsis group. **(B)** A representative immuno blot displaying Complexes I-V Protein expression levels and HSP 60 (loading control). Complexes protein densities was quantified using Syngene –Gene Tools analysis software and expression levels was normalized with HSP60 and data presented in percentage. Values are mean ± S.D. (*n* = 4#/group). #represents pooled samples.

**Figure 4:**
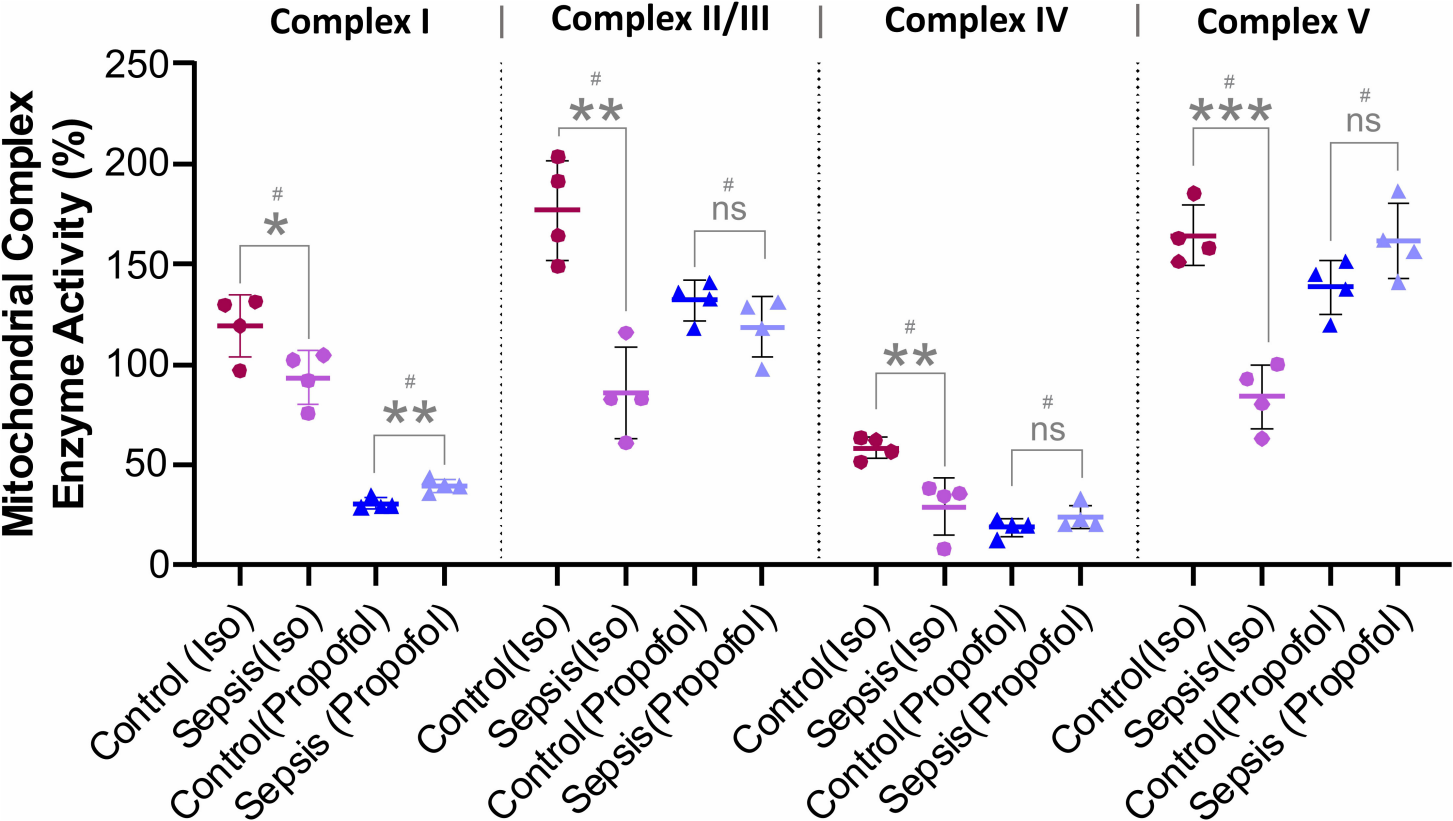
Effect of Sepsis with Isoflurane or Propofol anesthesia on Liver Mitochondrial respiratory chain complex (I, II/III, IV, and V) enzyme activity. Compared to controls isoflurane-sepsis group showed a significant reduction in enzymatic activities of complex-I (*p = 0.047), complex-II/III (**p = 0.001), complex-IV (**p = 0.008), and complex-V (***p = 0.0003). In Propofol sepsis group, except Complex I (**p = 0.005), no significant differences were observed in activities of Complexes II/III, IV, and V. Activity rates are expressed as percentage (%) of the control which had no sepsis. Values are mean ± S.D. (*n* = 4#/group). #represents pooled samples.

**Figure 5:**
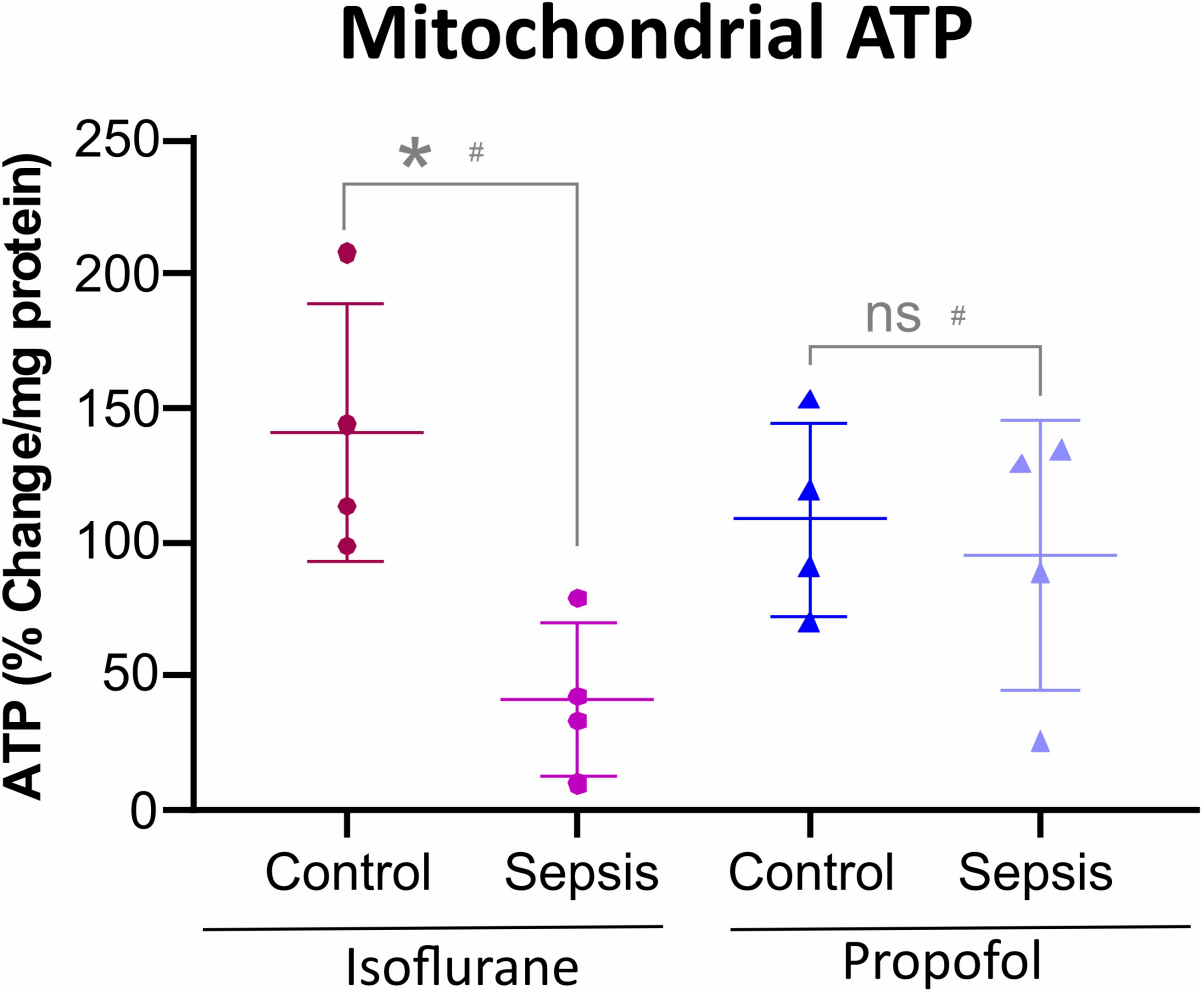
Anesthesia-Sepsis-associated changes in liver Mitochondrial ATP levels. Animals anesthetized with isoflurane-sepsis showed decreased mitochondrial ATP (adenosine triphosphate) levels (p* = 0.012), compared to controls, by Bioluminescent Assay. Whereas, animals anesthetized with Propofol-sepsis, showed no significant change (*p* = 0.670) in mitochondrial ATP content. *n* = 4# animals/group; Bars are mean ± S.D. #represents pooled samples.

**Figure 6:**
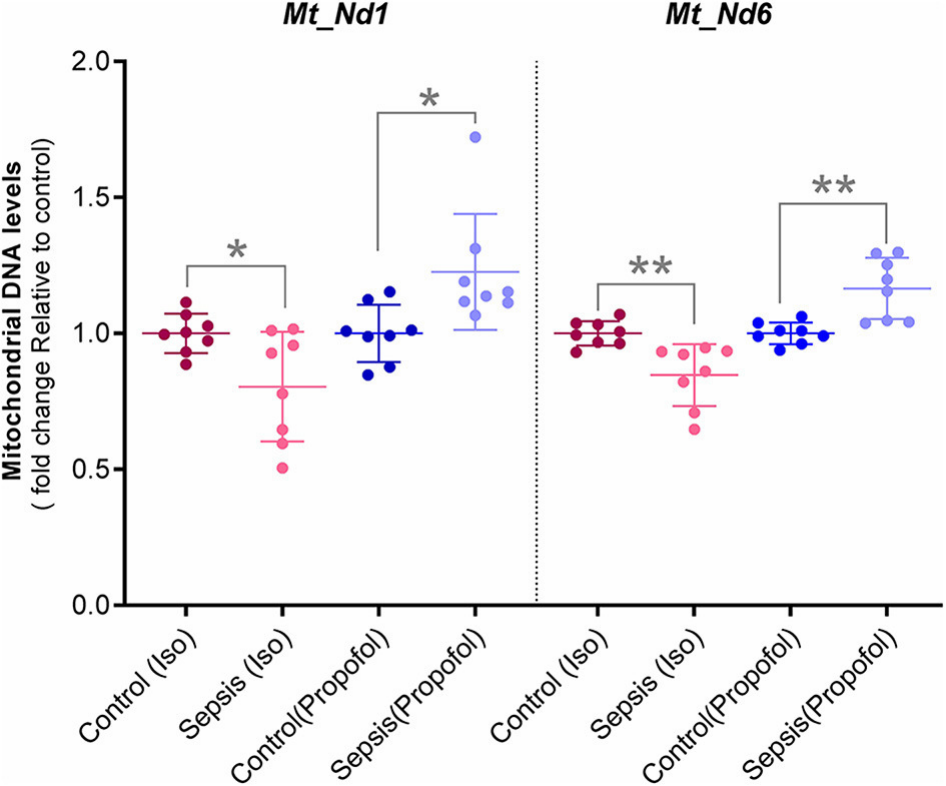
Mitochondrial DNA levels quantified with qPCR: Two Mitochondrial DNA encoded genes (Mt-Nd1 and Mt-Nd6) levels was normalized to a nuclear gene (Tubulin A1) and relative mtDNA levels was expressed as fold change to controls. Decreased mtDNA at gene Mt-Nd1 (p* = 0.040) and gene Mt-Nd6 (p** = 0.003) was observed in Isoflurane +Sepsis group compared to controls which had no sepsis. Interestingly, a small increase in mtDNA, Mt-Nd1 (p** = 0.007), and Mt-Nd6 (p** = 0.003) was seen in Propofol-sepsis group. *n* = 7–8 animals/group; Bars are mean ± S.D.

**Figure 7:**
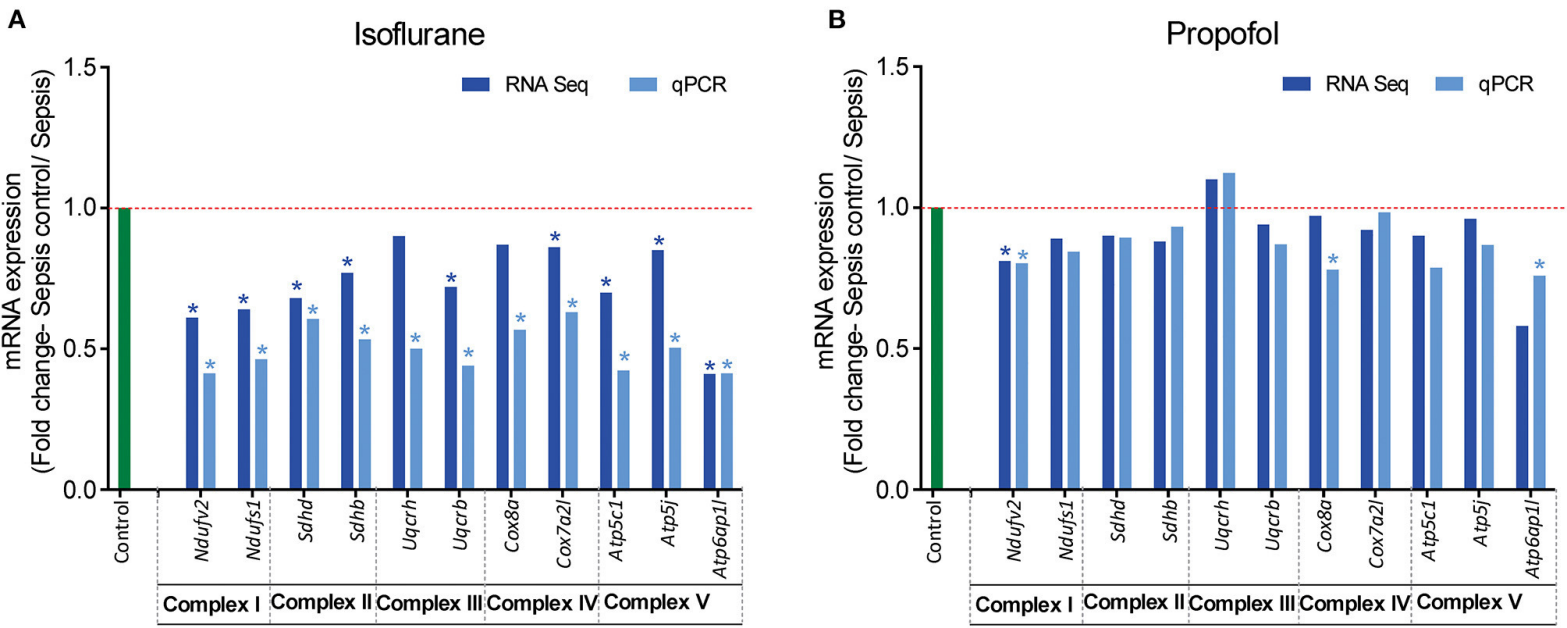
RT-qPCR validation of mitochondrial OXPHOS complex (I-V) genes that showed altered expression in RNASeq analysis. Fold change of gene expression is represented for **(A)** Isoflurane sepsis, **(B)** Propofol sepsis. From the RNA-Seq mRNA expression profiles, 2–3 genes with altered expression, encoding in mitochondrial OXPHOS complexes (I-V) were selected and gene expression was verified with RT-qPCR. Isoflurane sepsis effects the expression of all genes representing the 5 mitochondrial oxidative phosphorylation complexes (I-V). All genes represented here are significantly down-regulated compared to normal (9/11 genes show significant difference with both RNASeq and qPCR). However, for Propofol-sepsis group there is no significant change compared to normal, as determined by both mRNA-RNA-Seq and RT-qPCR analysis (only 1/11 genes show significant difference with both methods). The results of RT-qPCR are consistent with those of RNA-Seq mRNA expression profiles. “*” denotes significant (*p* < 0.05) expression obtained with RT-qPCR and for RNA-Seq it is the FDR adjusted *P*-value for differential expression. The red dashed line represents the control group fold change level (FC = 1) and green bar represents the RT-qPCR control group.

**Figure 8:**
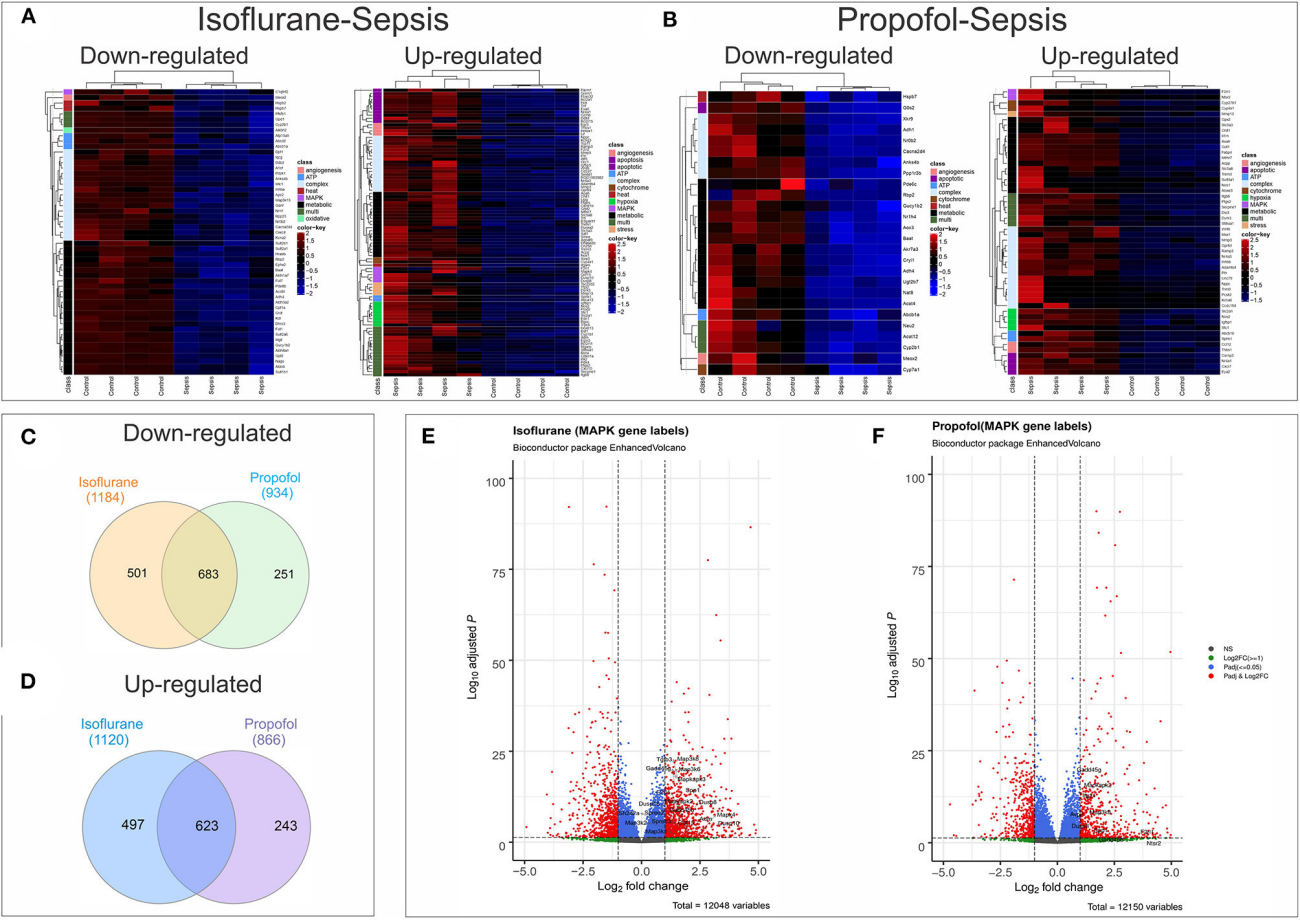
Anesthesia-Sepsis-associated alterations in liver gene expression profiles. Heatmap of genes* that were differentially expressed (adjusted *P*-value or Benjamini-Hochberg (BH) adjustment < 0.05; log2 fold change> 2 for up-regulated and log2 fold change < -2 for down-regulated genes) during sepsis under **(A)** Isoflurane background **(B)** Propofol background **(C)** Venn diagram showing number of common and unique genes downregulated (adjusted *P* < 0.05) in sepsis with Isoflurane and Propofol backgrounds. No cut off for log2 fold change was used to avoid any bias in representing the gene counts that are unique for only one group. **(D)** Venn diagram showing number of common and unique genes up-regulated (adjusted *P* < 0.05) in sepsis with Isoflurane and Propofol backgrounds. No cut off for log2 fold change was used. **(E)** Volcano plot for differentially expressed genes in sepsis with a Propofol background. Genes related to MAPK that were significant (adjusted *P* < 0.05 and log2 fold change> 1) are labeled in the plot. **(F)** Volcano plot for differentially expressed genes in sepsis with an Isoflurane background. Genes related to MAPK that were significant (adjusted *P* < 0.05 and log2 fold change> 1) are labeled in the plot. *Genes displayed in the heatmap are differentially expressed genes (adjusted *P* < 0.05) that had gene description lines like-angiogenesis, cytochrome, oxidative, stress, hsp, electron, heat, atp, metabolic, apoptotic/apoptosis, mitochondria, hypoxia, complex, mapk, kappa, and nfk pathways. For genes that matched more than one of these terms were grouped under “multi”.

**Figure 9:**
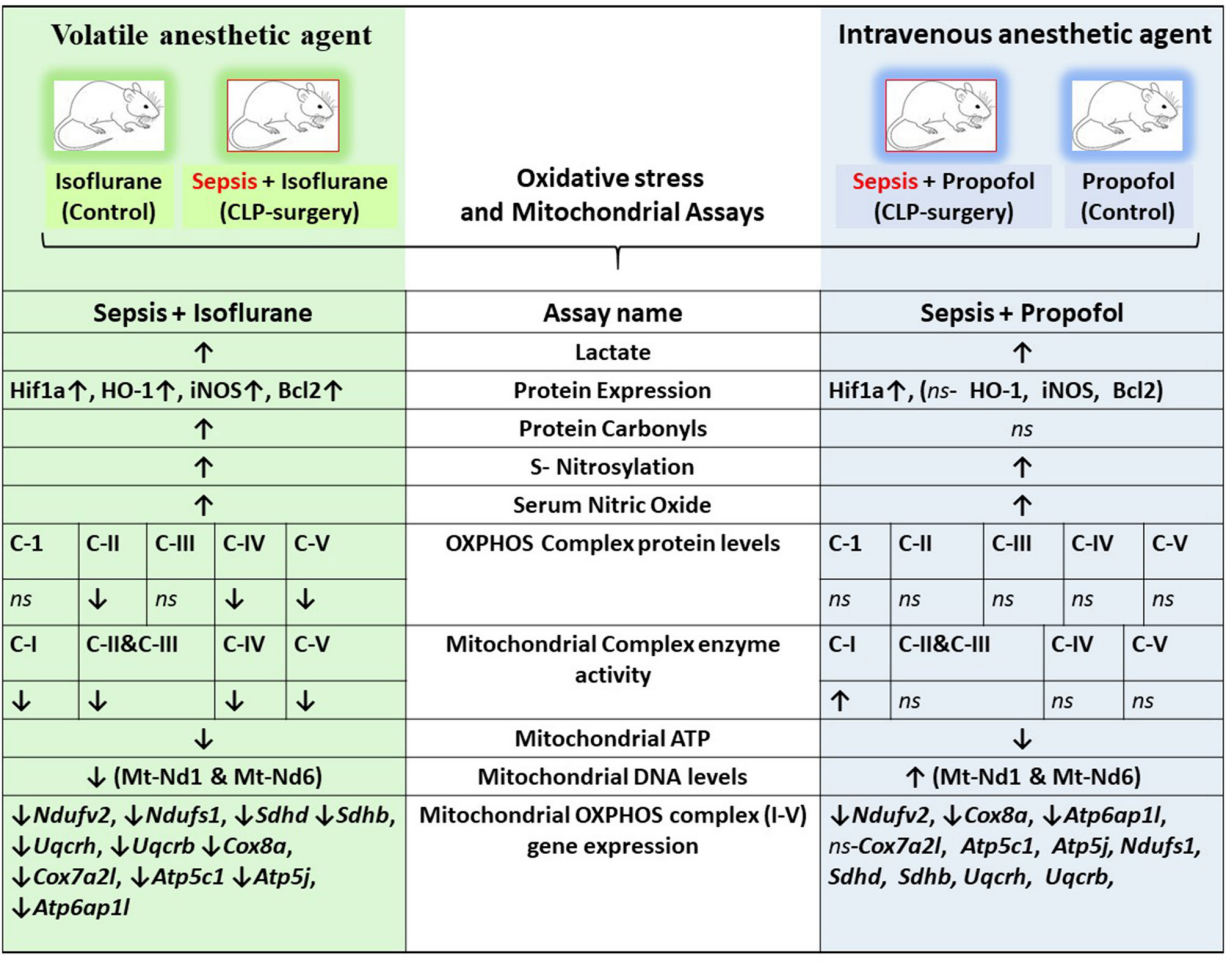
Integrative summary of the major observed effect of the Sepsis with Isoflurane or Propofol anesthesia on rat liver gene expression and subcellular energetics. ↑, increased; ↓, decreased; *ns*, not significant/no change; *C I-V*, Complex I-V; *Mt-Nd1*, mitochondrially encoded NADH dehydrogenase 1; *Mt-Nd6*, mitochondrially encoded NADH dehydrogenase 6; *Hif1a*, Hypoxia-inducible factor 1-alpha; *HO-1*, Heme oxygenase-1; *iNOS*, Inducible nitric oxide synthase; *Bcl2*, B-Cell CLL/Lymphoma 2; *Ndufv2*, NADH: Ubiquinone Oxidoreductase Core Subunit V2; *Ndufs1*, NADH:Ubiquinone Oxidoreductase Core Subunit S1; *SDHD*, Succinate Dehydrogenase Complex Subunit D; *SDHB*, Succinate Dehydrogenase Complex Iron Sulfur Subunit B; *UQCRH*, ubiquinol-cytochrome c reductase hinge protein; *UQCRB*, Ubiquinol-Cytochrome C Reductase, Complex III Subunit VI; *COX8a*, cytochrome c oxidase subunit 8A; *Cox7a2l*, cytochrome c oxidase subunit 7A2 like; *ATP5c1*, ATP synthase, H+ transporting, mitochondrial F1 complex, gamma polypeptide 1; *Atp5j*, ATP synthase, H+-transporting, mitochondrial F0 complex, subunit F; *Atp6ap1l*, ATPase H+ transporting accessory protein 1 like.

Overall, we found that compared to the control group (Anesthesia—Isoflurane or Propofol) + sham surgery (no CLP/sepsis/inflammation), rats anesthetized with isoflurane + CLP (sepsis/inflammation) group responded differently than animals anesthetized with Propofol + CLP (sepsis/inflammation). Group- specific differences are described in Figures 1–8 and a summary of major differences are shown in Figure 9. Hereafter the control group is referred to as Isoflurane or Propofol and the CLP- surgery group would be referred to as Isoflurane+ sepsis or Propofol+ sepsis.

### Cecal Ligation and Puncture (CLP) Induced Sepsis With Isoflurane and Propofol Expressed Different Levels of Illness Severity Markers

Mean arterial pressure (MAP), intravenous fluid requirements, relevant laboratory values (lactate, base deficit, glucose, creatinine, blood urea nitrogen [BUN]), as well as interleukin data are published in our recent publication (23). In both groups (isoflurane and Propofol), exposure to cecal ligation and puncture (CLP)/sepsis-inflammation led to increased fluid requirements, lower MAP at 8 h, development of a base deficit, and increased interleukins (IL-6, IL-1β, and TNF-α) at 8 h.

In the isoflurane group, exposure to sepsis-inflammation was associated with an increase in lactate (+ 0.64 mEq/L vs. −0.80 mEq/L, *p* = 0.0061); whereas in the Propofol group, it was not (−0.26 vs. −0.21 mEq/L, *p* = 0.84). Immediately following induction of anesthesia (prior to incision), rats in the isoflurane group had increased lactate compared to the rats in the Propofol group (23).

### Anesthesia and Sepsis-Inflammation Effects the Liver Hif1a, HO-1, Inos, and Bcl-2 Protein Expression Levels

Emerging data suggest that in the setting of Sepsis or inflammation, certain anesthetic exposures upregulate the HIF-1α (Hypoxia-inducible factor-1α) (23, 29) a key mediator of oxygen homeostasis and HIF-1 target proteins, involved in hepatic-inflammation (sepsis), and cause mitochondrial dysfunction and oxidative stress (30–33). In this study by using western blotting, Liver HIF-1α, HO-1 (heme oxygenase-1), iNOS (inducible nitric oxide synthase), and bcl-2 (B cell lymphoma 2) protein levels were examined (results shown in Figure 1). Compared to the control group (Isoflurane or Propofol + no sepsis), in rats anesthetized with isoflurane +sepsis led to a significant increase in the hepatic protein expression of HIF-1α (51%) as well as in Propofol +sepsis group (47%). However, in rats anesthetized with Propofol+sepsis, inflammation led to no significant difference in the hepatic expression of HO-1, iNOS, and bcl-2 proteins. In contrast, in rats anesthetized with isoflurane +sepsis, inflammation led to significant increases in the hepatic expression of HO-1 (58%), iNOS (92%), and bcl-2 (74%) proteins.

### S-Nitrosylation, Protein Carbonyl Content, and Serum Nitric Oxide Levels Was Increased With Sepsis-Isoflurane

Protein oxidation plays a significant role in physiological regulation, tissue injury, disease process, and also plays a crucial role in sepsis progression. The most commonly used marker to assess protein oxidation levels in tissue is by estimating protein-bound carbonyl levels. As shown in Figure 2, animals anesthetized with isoflurane and exposed to sepsis- exhibited increased expression of protein carbonyls (9.88 vs. 7.0 nM/mg protein) as well as S-nitrosylation (41% increase). In contrast, in animals anesthetized with Propofol +Sepsis inflammation led to an increase in S-nitrosylation (48%), but no significant change in expression of protein carbonyls was observed. In both anesthetic groups exposure to sepsis led to increases in serum nitric oxide levels (Isoflurane +sepsis, 48.560 (μM) and Propofol +Sepsis, 51.522 (μM), respectively.

### Mitochondrial OXPHOS Complex Protein Levels Are Decreased in Sepsis-Isoflurane

Mitochondrial respiratory chain contains five enzyme complexes that generate ATP by oxidative Phosphorylation (OXPHOS). As shown in Figure 3, compared to controls, in Isoflurane + Sepsis animals liver mitochondrial OXPHOS proteins expression levels were decreased significantly at Complex-II (39%), Complex-IV (32%), and at Complex-V (35%). Complex–I & III proteins were not significantly changed in the sepsis-isoflurane group. However, in animals anesthetized with Propofol, exposure, and sepsis-inflammation, did not show any significant detectable changes in the mitochondrial Complex-I-V protein expression levels.

### Isoflurane and Sepsis-Inflammation Decrease the Liver Mitochondrial Complexes Enzyme Activity

As shown in Figure 4, compare to the controls, in animals anesthetized with isoflurane, exposure to sepsis-inflammation led to significant decreases in liver mitochondrial complex activity at Complex I (26%), Complex II/III (86%), Complex IV (29%), and Complex V (80%). In contrast, in animals anesthetized with Propofol, exposure to sepsis-inflammation led to no significant change in mitochondrial complex activity at Complex II/III, IV, and Complex V. However, we observed a small percent (9%) increase of complex I activity in Propofol-sepsis group.

### Isoflurane and Sepsis-Inflammation Reduces the Liver Mitochondrial ATP Levels

Both Oxidative phosphorylation (OXPHOS) and glycolysis place a substantial contribution to ATP production in cells. Inhibition or reduction of OXPHOS complexes results in a considerable decrease in ATP levels. As shown in Figure 5, in animals anesthetized with isoflurane, sepsis induced inflammation significantly decreased the mitochondrial ATP (42%) compared to control group. In contrast, Propofol + sepsis liver mitochondrial ATP levels had no significant change (14%) compared to controls.

### Isoflurane and Sepsis Decrease the Liver Mitochondrial DNA Encoded Genes (Mt-Nd1 and Mt-Nd6)

Mitochondrial DNA (mtDNA) codes for the mitochondrial respiratory chain complex (I-V) core subunits, and change in mitochondrial DNA encoded protein levels results, in disruption of mitochondrial respiratory chain complexes biogenesis (34).

In these studies, we observed decreased OXPHOS complex protein levels along with their complex enzyme activity. Here, the qPCR quantified Hepatic Mitochondrial DNA levels of Mt-ND1 (mitochondrial encoded NADH-ubiquinone oxidoreductase core subunit-1) and Mt-ND6 (mitochondrial-encoded NADH-ubiquinone oxidoreductase core subunit-6) along with Tubulin, (nuclear DNA) are shown in Figure 6. Compare to controls, in animals exposed to isoflurane+ Sepsis, Mt_Nd1 (1 vs. 0.84-fold) and Mt_Nd6 (1 vs. 0.87-fold) mitochondrial DNA levels were significantly decreased. In contrast, animals exposed to Propofol + sepsis, liver mitochondrial DNA levels at Mt_Nd1 (1 vs. 1.15-fold) and Mt_Nd6 (1 vs. 1.14-fold) were slightly increased compared to control.

### Isoflurane and Sepsis Decreases the Expression of Genes Regulating Mitochondrial OXPHOS Complexes and Associated Pathways

Analysis of mRNA expression for genes specific to Complex I-V of the electron transport chain revealed that in animals anesthetized with isoflurane, exposure to inflammation produces a consistent decrease in mRNA for all five complexes (confirmed with quantitative PCR). In contrast, in animals anesthetized with Propofol, exposure to inflammation produced no significant changes in mRNA for any of the five complexes (Figure 7). All statistically significant up and down-regulated genes related to the mitochondria or oxidative phosphorylation as identified by differential expression analysis of RNA sequencing data are presented in Figure 8, and Supplementary Table 1 in Supplemental Digital Content (Supplementary Tables 2, 3). As is evident from Supplementary Figure 1, the number of OXPHOS genes that are differentially expressed compared to controls is higher in Isoflurane-Sepsis (S1 A) compared to the Propofol-Sepsis group (S1 B), and are down-regulated.

Screening of genes based on term matches in gene descriptions (as mentioned in methods section) from the RNASeq differential expression results (Adjusted *P* < 0.05 and log2FC >2 or < -2) showed that more genes were perturbed in the Isoflurane-Sepsis group compared to Propofol-Sepsis (Figures 8A,B). Mitogen Activated Protein kinases (MAPK) have roles as mediators of inflammation and MAPK signaling is known to be enhanced following oxidative stress (35, 36). At a log2FC>2 (adjusted *P* < 0.05) (Figure 8A) MAPK related genes F2rl1, Adm (marked as multi, as it was picked up for term searches “metabolic,” “hypoxia,” and “MAPK”), Mapk4, Gdf15, Dusp10, and Dusp8 were up-regulated in Isoflurane-Sepsis compared to only 2 genes (Figure 8B) in the Propofol-Sepsis group. The number of up-regulated MAPK genes is more (20 genes) for Isoflurane-Sepsis group, at a more relaxed log2FC of > 1 (Figure 8E) as compared to a fewer genes in Propofol-Sepsis group (Figure 8F). While the cell death pathway/apoptosis related genes are mostly up-regulated in the Isoflurane-Sepsis group (11 genes), the Propofol-Sepsis group shows an up-regulation of fewer genes (4 genes) and down regulation of a single gene (G0s2) which is predicted to be involved in extrinsic apoptotic signaling pathway (Figures 8A,B). Other related genes that are up and down-regulated under the two anesthetic conditions along with their associated terms are shown in Figures 8A,B. We also looked at the total number of genes that were up and down-regulated (adjusted *P* < 0.05) for the two groups in our study for the genes that were picked by our gene term searches (search terms as in Figures 8A,B), but without setting a cut-off for log2FC values. This data is represented in Figures 8C,D. As seen from the figure, many genes are common to both groups, yet the unique number of genes that are differentially expressed is more for the Isoflurane-Sepsis group. A KEGG pathway enrichment analysis with all the differentially expressed genes at a significant cut-off for adjusted *P* < 0.05 and *q*-value = 0.10, showed PI3K-Akt signaling and MAPK signaling as the top 2 pathways (i.e., highest number of genes) for the Isoflurane-Sepsis group (Supplementary Figure 2A). With the Propofol-Sepsis group we do not see these pathways in the top 50 list. However, one of the top pathways was Rap1 signaling (which is also seen in the Isoflurane-Sepsis group). It is interesting that RAP1 has roles in regulating MAPK kinase activity (Supplementary Figure 2B).

## Discussion

Sepsis is defined as “life-threatening organ dysfunction caused by a dysregulated host response to infection” (37) and is mostly characterized by a simultaneous occurrence of immunosuppressive and hyper-inflammatory reactions. In sepsis development, PAMPs (Pathogen-associated molecular patterns) play a key role in the inflammatory response and particularly in the hyper inflammatory reaction (38, 39). Hepatic dysfunction plays a particularly prominent role in septic outcomes, increasing long term mortality by more than any other organ system with the exception of the central nervous system (40). A comprehensive understanding of the impact of ICU interventions on hepatic function is essential to maximizing clinical outcomes.

A growing body of both animal and human data suggest that VAAs (e.g., Isoflurane) may improve survival and organ function after exposure to inflammation and sepsis (11, 12, 14). While the mechanisms of APC in neurologic or myocardial protection have been well-described (41) however, anesthetics- protective mechanisms in the setting of inflammation and sepsis are not well-understood. Anesthetic preconditioning (APC) activates the myocardial K_ATP_ channels, protein kinase C, and leads to the generation of oxidative species in addition to altering the mitochondrial permeability transition pore (41). In non-septic animals, metabolic differences between VAAs and GABA agonists have been identified (19, 42).

Sepsis likely has an impact on energy production at the level of the mitochondria, most likely at longer time points and with higher disease severity (43, 44) although the aggregated data from various animal models, tissues, and time points are to some extent conflicting (45). Previous authors have found evidence that VAAs inhibit Complex I of the ETC, favoring anaerobic metabolism (and lactate production) (19, 20). Our results demonstrated higher lactate values *prior* to incision (2.13 vs. 1.12 mEq/L, *p* < 0.0001, both groups), after sham surgery (1.88 vs. 0.851 mEq/L, *p* < 0.0001), and after CLP (2.48 vs. 0.846 mEq/L, *p* = 0.0002), when compared to rats anesthetized with Propofol. This is consistent with prior work. Importantly, isoflurane has been demonstrated to have hepatotoxic effects when given at high doses [e.g., 2% (46); of note we used 1.4% isoflurane]. Because the liver plays a key role in lactate clearance, it is possible that these observations are also due, to some extent, to hepatic dysfunction.

While both the isoflurane and Propofol groups exhibited an upregulation in HIF-1 with exposure to sepsis, only the isoflurane group exhibited upregulation in HO-1 [an anti-oxidant protein that produces carbon monoxide and initiates mitochondrial biogenesis (47)], iNOS [an enzyme that produces the free radical NO under conditions of oxidative stress, and which is known to be hepatotoxic (48)], and bcl-2 [a key regulator of mitochondrial autophagy, which inhibits beclin-1 (49)]. These protective responses can be a sign of stress (e.g., hepatotoxicity) but may also manifest as improved outcomes—this will require a survival study to determine (which is ongoing).

Mitochondria is the powerhouse of the cell and has vital roles in energy production, calcium homeostasis, and programmed cell death. In addition, it is known to be involved in antibacterial, antiviral, and several stress responses to hypoxia and tissue injury (50–52). Most interestingly, in our study of animals anesthetized with isoflurane, electron transport chain complex activity was diminished, mitochondrial OXPHOS protein levels were decreased, and mitochondrial ATP levels were lowered following exposure to sepsis, suggesting decreased hepatic energy production (which would be consistent with a reduction in hepatic lactate metabolism). In contrast, animals anesthetized with Propofol exhibited very little change in either electron transport chain complex activity or OXPHOS protein levels following exposure to sepsis, with no change in mitochondrial ATP levels. Interestingly, when comparing anesthetic agents (both before and after sepsis), we notice significant reduction in Complex I and IV *activity* with Propofol. This is in distinction to Makaryus et al.'s findings which primarily identified inhibition of Complex II, although they utilized pMRS and were focused on the brain, not the liver. Our results are consistent with other work in rats that has shown a clear reduction in hepatic cytochrome c reductase (Complex II/III) activity in rats infused with Propofol (53).

Animals in both groups (isoflurane and Propofol) exhibited evidence of increased *nitrosative* stress in both tissue (S-nitrosylation) and serum (NO) following exposure to inflammation. In contrast, in animals anesthetized with isoflurane, inflammation led to an increase in *oxidative* stress in the liver, whereas in animals anesthetized with Propofol, inflammation led to a decrease in oxidative stress in the liver. Prior work has suggested that Propofol may prevent oxidative stress during neuronal excitotoxicity (54) as well as ischemia-reperfusion injury models (22, 55), and there is some animal data suggesting that sepsis survival is lower when Propofol is used (as compared to volatile anesthetic agents). Because of the liver's prominent role in affecting long term sepsis mortality and the known impact of Propofol on hepatic cytochrome c reductase activity in rats (53), better understanding the impact of sepsis on hepatic subcellular energetics and in particular oxidative stress is important.

Consistent with our OXPHOS and ATP levels, we found that mitochondrial encoded gene DNA levels were decreased [Mt_Nd1 (1 vs. 0.84-fold) and Mt_Nd6 (1 vs. 0.87-fold)] in rats anesthetized with isoflurane and exposed to sepsis, but slightly increased in those anesthetized with Propofol and exposed to sepsis (Figure 6). Together, these data strongly suggest that VAAs result in a programmatic decrease in mitochondrial activity.

Differential expression analysis of RNA-Seq data suggests that genes related to energy metabolism, cellular protection, and oxidative stress are altered more in the Isoflurane-sepsis group (Figure 8A) compared to Propofol-sepsis group (Figure 8B). Also, RNASeq and qPCR-mRNA expression data from liver samples suggests that the response to sepsis-inflammation may differ in animals anesthetized to isoflurane, as compared to animals anesthetized with Propofol (Supplemental Digital Content, Supplementary Tables 2, 3, Supplementary Figure 1) and Figure 7. In particular, in animals anesthetized with isoflurane, inflammation led to upregulation of 6 genes related to MAPK [an important mediator of inflammation during infection (35, 56, 57)], whereas in animals anesthetized with Propofol, sepsis induced inflammation led to upregulation of 2 genes related to MAPK (Figure 8). This number was still higher when compared at a more relaxed log2FC cut off value. To note, the MAPK pathway was one of the top 50 enriched pathways for Isoflurane-sepsis group. Despite that difference, we observed similar increases in serum interleukins with inflammation in both groups (isoflurane and Propofol). Our model, which only allowed 8 h of sepsis exposure, may not have allowed enough time for differences in mRNA expression to manifest (see Limitations, below). Given that sepsis outcomes are strongly influenced by the balance between pro- and anti-inflammatory responses (referred to as the “sepsis seesaw”) that change over a period of days (58), substantial differences in the early immunological response to infection may explain some of the reported differences in outcomes reported by groups comparing volatile anesthetic gases to GABA agonists (9–14).

The primary significance of our results relates to hepatic function in the setting of infection, which is a key driver of clinical outcomes in humans (as described above). The use of VAAs (as opposed to GABA agonists) in the setting of sepsis clearly impacts anaerobic metabolism, lactate clearance, or both. Whether or not this explains the improved outcomes that other investigators have identified when VAAs are used in both animal models of sepsis as well as humans, requires further investigation.

Our study has limitations. First, it was conducted in rats. Thus, our findings are not necessarily generalizable to humans. Second, Propofol is a lipid emulsion, the lipid component of which may have its own impact on metabolism. Third, we utilized 100% oxygen as part of our anesthetic which is relevant in the sickest patients but not necessarily applicable to those ventilated with 60% oxygen or less. Sepsis is a dynamic disease process over time, thus our findings likely reflect early phase of the disease. Bacteria concentration in blood peak between 6 and 12 h after performing cecal ligation (59, 60), and we harvested the tissue at 8 h. mRNA data, which looks at transcript expression of all genes in a tissue, is less susceptible to confirmation bias (because thousands of genes are analyzed), but requires confirmation with various approaches before any mechanistic pathways can be elucidated with confidence. Nevertheless, our study provides initial data to design those mechanistic studies. Lastly, the focus of this study was on the liver, which is rapidly affected by the CLP model [nitric oxide levels peak within ~2 h (61)]—our findings may not be applicable to other organ systems, such as the central nervous or cardiovascular systems.

## Conclusions

We conclude that rats anesthetized with isoflurane and Propofol respond differently to inflammation initiated by cecal ligation and puncture (Figure 9). Isoflurane anesthesia leads to lactic acidosis as compared to Propofol—it is not clear whether this is due to an increase in anaerobic metabolism or a decrease in hepatic clearance of lactate. Rats anesthetized with isoflurane respond to inflammation with increase in hepatic HO-1, iNOS, and bcl-2, whereas rats anesthetized with Propofol do not. While rats anesthetized with isoflurane and Propofol exhibit hepatic nitrosative stress during sepsis-inflammation, increased tissue oxidative stress was only noted in rats anesthetized with isoflurane. Rats anesthetized with isoflurane respond to inflammation with decrease OXPHOS protein expression and decreased ETC Complex activity, whereas rats anesthetized with Propofol do not. The combination of isoflurane and inflammation may lead to over-expression of mRNA involved in the pro-inflammatory MAPK pathway and many other pathways associated with sepsis-inflammation (Supplementary Figure 2A). More work is needed to determine whether or not these Anesthesia-Sepsis-associated differences in the mitochondrial metabolic response to inflammation produce differences in organ protection or animal survival.

## Data Availability Statement

All processed data were uploaded to the gene expression omnibus (https://www.ncbi.nlm.nih.gov/geo/; accession # GSE138454).

## Ethics Statement

The animal study was reviewed and approved by animal care and use committee (IACUC) at the University of Virginia (protocol 4140).

## Author Contributions

RT: design of study, conduct of study (animal work), interpretation of data, and writing manuscript. HO: conduct of study (biochemical work), interpretation of data, writing manuscript, and review of manuscript. UP: RNA sequencing analysis, interpretation of data, and review of manuscript, KI: construction of NIRS equipment and conduct of study (animal work). ZZ: design of study, interpretation of data, and review of manuscript. All authors contributed to the article and approved the submitted version.

## Conflict of Interest

The authors declare that the research was conducted in the absence of any commercial or financial relationships that could be construed as a potential conflict of interest.

## Abbreviations

CLP: cecal ligation and puncture
BCA: bicinchoninic acid
DNPH, 2: 4-dinitrophenylhydrazine
DPF: diferential pathlength factor
ETC: electron transport chain
ETCO2: end tidal CO2
IPC: ischemic preconditioning
MMTS: methyl methanethiosulfonate
NIRS: near infrared spectroscopy
NS: normal saline
CytOX: oxidation state of cytochrome aa3
PBS: phosphate bufer saline
TCA: tricyclic acid
TNF: tumor necrosis factor
VAAs: volatile anesthetic agents
*mEq/L*: milliequivalents Per Liter (mEq/L)
*nM/mg*: nanomole/milligram
μ*M*: micromoles
*Mt-Nd1*: mitochondrially encoded NADH dehydrogenase 1
*Mt-Nd6*: mitochondrially encoded NADH dehydrogenase 6
*Hif1a*: Hypoxia-inducible factor 1-alpha
*HO-1*: Heme oxygenase-1
*iNOS*: Inducible nitric oxide synthase
*Bcl2*: B-Cell CLL/Lymphoma 2
*Ndufv2*: NADH: Ubiquinone Oxidoreductase Core Subunit V2
*Ndufs1*: NADH:Ubiquinone Oxidoreductase Core Subunit S1
*SDHD*: Succinate Dehydrogenase Complex Subunit D
*SDHB*: Succinate Dehydrogenase Complex Iron Sulfur Subunit B
*UQCRH*: ubiquinol-cytochrome c reductase hinge protein
*UQCRB*: Ubiquinol-Cytochrome C Reductase Complex III Subunit VI
*COX8a*: cytochrome c oxidase subunit 8A
*Cox7a2l*: cytochrome c oxidase subunit 7A2 like
*ATP5c1*: ATP synthase, H+ transporting, mitochondrial F1 complex gamma polypeptide 1
*Atp5j*: ATP synthase, H+-transporting, mitochondrial F0 complex subunit F
*Atp6ap1l*: ATPase H+ transporting accessory protein 1 like.
APC: anesthetic preconditioning
bcl-2: B-cell lymphoma 2.
