# Supplementary material for: Anesthesia-Sepsis-Associated Alterations in Liver Gene Expression Profiles and Mitochondrial Oxidative Phosphorylation Complexes
Source: Front Med (Lausanne). 2020 Dec 18;7:581082. doi: 10.3389/fmed.2020.581082 (PMC7775734; doi:10.3389/fmed.2020.581082)
Supplement: Supplementary file 1 [file Table_1.docx]

**Supplementary Table 1. Animal tissue pooling details for Liver mRNA analysis and OXPHOS protein expression**.

| **Treatment group** | **Number of samples** | **Pooling per each sample** | **Total number of rats used** |
| --- | --- | --- | --- |
| Propofol: Control | 4 | 3 | 12 (4 x 3) |
| Propofol: Sepsis/CLP | 4 | 3 | 12 (4 x 3) |
| Isoflurane: Control | 4 | 3 | 12 (4 x 3) |
| Isoflurane: Sepsis/CLP | 4 | 3 | 12 (4 x 3) |

Note: We used pooled tissue samples only for RNA Sequence and for mitochondrial analysis because it is cost-effective. We felt that our sampling plan (RNA Sequence studies) balanced cost with results. We believe that a pooling experimental design is more efficient than a random sample strategy (Schisterman and Vexler 2008; Urena-Peralta et al. 2018; Goodall et al. 2019) although we acknowledge that some granularity is lost.
