# Supplementary material for: Anesthesia-Sepsis-Associated Alterations in Liver Gene Expression Profiles and Mitochondrial Oxidative Phosphorylation Complexes
Source: Front Med (Lausanne). 2020 Dec 18;7:581082. doi: 10.3389/fmed.2020.581082 (PMC7775734; doi:10.3389/fmed.2020.581082)
Supplement: Supplementary file 2 [file Table_2.docx]

**Supplementary Table 2 differentially expressed nuclear encoded OXPHOS genes in liver sepsis.**

The excel tables list the differentially expressed genes in liver sepsis model (sepsis + anesthesia) of Rat liver (at a significant adjusted P-value cut off of less than 0.05) compared to controls (no sepsis + anesthesia) with the two anesthetic backgrounds (Propofol and Isoflurane).

| **Table 2: a) Nuclear encoded OXPHOS genes that were significantly differentially expressed (adjusted P-value < 0.05) following sepsis in the rat liver compared to control (no sepsis + isoflurane) in an Isoflurane background** | |  | |  |  |
| --- | --- | --- | --- | --- | --- |
| **Liver sepsis model ( Isoflurane)** | |  | |  |  |
| **Gene symbol** | | **log2 fold change** | | adjusted P-value |  |
| **Respiratory chain complex I** | |  | |  |  |
| Ndufa10 | | -0.430885536 | | 0.01557882 |  |
| Ndufa11 | | -0.26794455 | | 0.02862335 |  |
| Ndufa12 | | -0.434655785 | | 2.59E-02 |  |
| Ndufa8 | | -0.753754358 | | 1.18E-05 |  |
| Ndufa9 | | -0.387939373 | | 1.10E-03 |  |
| Ndufab1 | | -0.460521488 | | 1.22E-05 |  |
| Ndufaf1 | | -0.633558723 | | 5.11E-06 |  |
| Ndufaf3 | | -0.724868354 | | 5.40E-05 |  |
| Ndufaf5 | | -0.442702682 | | 4.56E-02 |  |
| Ndufb10 | | -0.636132618 | | 3.93E-06 |  |
| Ndufb6 | | -0.492928495 | | 0.00074927 |  |
| Ndufb7 | | -0.461933719 | | 0.00422178 |  |
| Ndufb8 | | -0.272955079 | | 1.71E-02 |  |
| Ndufb9 | | -0.427674133 | | 7.87E-05 |  |
| Ndufc2 | | -0.418493601 | | 6.17E-06 |  |
| Ndufs4 | | -0.448210479 | | 3.07E-05 |  |
| Ndufs5 | | -0.283384135 | | 0.01417958 |  |
| Ndufs7 | | -0.519738979 | | 1.25E-04 |  |
| Ndufs8 | | -0.429441274 | | 7.03E-04 |  |
| Ndufv1 | | -0.283731848 | | 6.51E-04 |  |
| Ndufv3 | | -0.423201121 | | 2.59E-05 |  |
| Ndufs1 | | -0.646233313 | | 3.19E-06 |  |
| Ndufs3 | | -0.70089263 | | 6.00E-06 |  |
| Ndufv2 | | -0.716882647 | | 5.42E-08 |  |
| **Respiratory chain complex II** | |  | |  |  |
| Sdhaf2 | | -0.409905776 | | 2.96E-08 |  |
| Sdhc | | -0.458287536 | | 0.0004677 |  |
| Sdha | | -0.322162608 | | 0.00272936 |  |
| Sdhb | | -0.375634611 | | 7.92E-05 |  |
| Sdhd | | -0.549108834 | | 2.70E-06 |  |
| **Respiratory chain complex III** | |  | |  |  |
| Uqcrb | | -0.472502973 | | 1.55E-08 |  |
| Uqcrc2 | | -0.192834278 | | 3.84E-03 |  |
| **Respiratory chain complex IV** | |  | |  |  |
| Cox15 | | -1.133277701 | | 1.05E-11 |  |
| Cox20 | | -0.782522306 | | 1.84E-06 |  |
| Cox4i2 | | 1.973963357 | | 0.04482123 |  |
| Cox7a2l | | -0.210222959 | | 0.01420847 |  |
| **Respiratory chain complex V** | |  | |  |  |
| Atp5g3 | | -0.272382254 | | 0.00277479 |  |
| Atp5j | | -0.228342438 | | 0.010982 |  |
| Atp5l | | -0.142758496 | | 0.04563137 |  |
| **CoenzymeQ** | |  | |  |  |
| Coq2 | | -0.583314776 | | 1.25E-04 |  |
| Coq3 | | -0.812935376 | | 1.02E-10 |  |
| Coq4 | | -0.584720574 | | 0.02776274 |  |
| Coq5 | | -0.61970643 | | 0.0002247 |  |
| Coq7 | | -0.823646169 | | 2.11E-05 |  |
| Coq9 | | -0.822267596 | | 2.05E-14 |  |
| **Respiratory chain assembly factors** | |  | |  |  |
| Acad9 | | -0.933184204 | | 1.74E-07 |  |
| Atpaf1 | | -0.86124657 | | 6.57E-06 |  |
| Coa3 | | -0.324926534 | | 0.03147849 |  |
| Coa4 | | -0.958002018 | | 2.92E-07 |  |
| Cox19 | | -0.580716278 | | 2.75E-09 |  |
| Ecsit | | -0.612725196 | | 4.71E-06 |  |
| Ndufaf5 | | -0.442702682 | | 0.0455906 |  |
| Ttc19 | | -0.358783926 | | 0.00072735 |  |
| Oxa1l | | -0.204791884 | | 0.04015836 |  |
| Timm21 | | -0.57552242 | | 0.0019922 |  |
| Fastkd3 | | 0.317705578 | | 0.01306779 |  |
| Coa5 | | -0.214569257 | | 0.0004401 |  |
| Nubpl | | -0.569301489 | | 2.47E-03 |  |
| Taz | | -0.301616713 | | 0.02116212 |  |
| Tmem126b | | -0.693030503 | | 0.00014739 |  |
| Aifm1 | | -0.242742439 | | 0.00085416 |  |
| Lyrm7 | | -1.280197826 | | 0.00306577 |  |
| **Table 2: b) Nuclear encoded OXPHOS genes that were significantly differentially expressed (adjusted P-value < 0.05) following sepsis in the rat liver compared to control (no sepsis + propofol) in a Propofol background** |  | |  | | |
| **Liver sepsis model ( Propofol)** |  | |  | | |
| **Gene symbol** | **log2 fold change** | | **adjusted P-value** | | |
| **Respiratory chain complex I** |  | |  | | |
| Ndufa8 | -0.423066137 | | 0.000163535 | | |
| Ndufaf3 | -0.818431059 | | 4.64E-07 | | |
| Ndufaf4 | -0.518164644 | | 0.000772483 | | |
| Ndufb10 | -0.338816788 | | 0.014222118 | | |
| Ndufs2 | 0.237554792 | | 0.009907287 | | |
| Ndufs4 | -0.203697882 | | 0.048028895 | | |
| Ndufv3 | -0.406580927 | | 0.004166605 | | |
| Ndufv2 | -0.306497944 | | 0.01771462 | | |
| **Respiratory chain complex II** |  | |  | | |
| Sdhc | -0.170183328 | | 0.047397727 | | |
| **Respiratory chain complex III** |  | |  | | |
| Slc25a33 | -0.714514856 | | 6.91E-05 | | |
| **Respiratory chain complex IV** |  | |  | | |
| Cox15 | -0.572797624 | | 0.000381347 | | |
| Cox20 | -0.601104747 | | 0.00214129 | | |
| **Respiratory chain complex V** |  | |  | | |
| NA |  | |  | | |
| **CoenzymeQ** |  | |  | | |
| Coq10b | 1.626449426 | | 2.00E-13 | | |
| Coq2 | -0.45853748 | | 0.002131253 | | |
| Coq3 | -0.611938102 | | 1.61E-05 | | |
| Coq9 | -0.551856213 | | 0.000104355 | | |
| **Respiratory chain assembly factors** |  | |  | | |
| Acad9 | -0.384637684 | | 0.00227635 | | |
| Atpaf1 | -0.480928399 | | 0.000158808 | | |
| Coa4 | -0.639949271 | | 0.000391406 | | |
| Ecsit | -0.289374531 | | 0.036287679 | | |
| Ndufaf4 | -0.518164644 | | 0.000772483 | | |
| Ttc19 | -0.244230931 | | 0.014715403 | | |
| Bcs1l | -0.552316248 | | 0.000672213 | | |
| Nubpl | -0.423598298 | | 0.049086614 | | |
| Oxa1l | -0.23147158 | | 0.039941083 | | |
| Coa4 | -0.639949271 | | 0.000391406 | | |
