# Supplementary material for: Anesthesia-Sepsis-Associated Alterations in Liver Gene Expression Profiles and Mitochondrial Oxidative Phosphorylation Complexes
Source: Front Med (Lausanne). 2020 Dec 18;7:581082. doi: 10.3389/fmed.2020.581082 (PMC7775734; doi:10.3389/fmed.2020.581082)
Supplement: Supplementary file 3 [file Table_3.docx]

**Supplementary Table 3 differentially expressed nuclear encoded mitochondrial genes in liver sepsis.**

The excel tables list the differentially expressed genes in liver sepsis model (sepsis + anesthesia) of Rat liver (at a significant adjusted P-value cut off of less than 0.05) compared to controls (no sepsis + anesthesia) with the two anesthetic backgrounds (Propofol and Isoflurane).

| **Table 3: a) Nuclear genes encoding mitochondrial proteins that were significantly differentially expressed (adjusted P-value<0.05) following sepsis in the rat liver compared to control (no sepsis + isoflurane) in an Isoflurane background** |  |  |
| --- | --- | --- |
| **Gene** | **log2 fold change** | **adjusted P-value** |
| Mgarp | 2.0400718 | 0.0036524 |
| Cox4i2 | 1.97396336 | 0.04482123 |
| Slc25a29 | 1.71402994 | 9.43E-13 |
| Rfk | 1.59540727 | 7.40E-25 |
| Lamc1 | 1.33167152 | 4.39E-09 |
| Gls | 1.18346512 | 8.16E-12 |
| Mthfd2 | 1.1678181 | 1.28E-06 |
| Rab32 | 1.16423344 | 1.23E-08 |
| Pdp1 | 1.14225182 | 4.85E-13 |
| Pptc7 | 1.1403311 | 1.06E-08 |
| Txndc12 | 1.11176377 | 8.96E-07 |
| Slc25a30 | 1.03055754 | 2.22E-06 |
| Hk1 | 0.99839925 | 4.03E-06 |
| Tspo | 0.88677548 | 0.00281156 |
| Pdp2 | 0.84698834 | 0.00015622 |
| Aifm3 | 0.80956191 | 0.00083844 |
| Sqrdl | 0.60323649 | 0.04818386 |
| Gpd2 | 0.59965393 | 0.00904374 |
| Bcl2l13 | 0.5917841 | 0.0002312 |
| Akap10 | 0.54555473 | 4.32E-05 |
| Htra2 | 0.52288519 | 2.24E-05 |
| Rab24 | 0.50162483 | 0.00020074 |
| Dnlz | 0.46642826 | 0.00037055 |
| Bax | 0.4621548 | 0.00011016 |
| Bid | 0.43103829 | 0.00555788 |
| Fam136a | 0.41690266 | 0.00029097 |
| Bcl2l2 | 0.41089683 | 3.93E-05 |
| Adck4 | 0.39260203 | 1.12E-05 |
| Qtrt1 | 0.34203513 | 0.01237978 |
| Bnip3l | 0.32244622 | 0.03366807 |
| Guk1 | 0.31253913 | 5.02E-05 |
| Rps14 | 0.26629036 | 0.00187514 |
| Pdha1 | 0.26507872 | 0.01402176 |
| Rpl34 | 0.23816689 | 0.00525101 |
| Cisd2 | 0.23578 | 0.04906004 |
| Rps15a | 0.23188526 | 0.00164655 |
| Trmt10c | 0.22599874 | 0.01185336 |
| Rpl10a | 0.21709436 | 0.02720929 |
| Ifi27 | 0.15950134 | 0.00209677 |
| Nrd1 | 0.15870221 | 0.01358819 |
| Atp5l | -0.1427585 | 0.04563137 |
| Oxa1l | -0.2047919 | 0.04015836 |
| Mfn1 | -0.2166699 | 0.00674511 |
| Dlst | -0.2203721 | 3.72E-05 |
| Bloc1s1 | -0.2220135 | 0.04668548 |
| Atp5j | -0.2283424 | 0.010982 |
| Prelid1 | -0.2385728 | 0.03335096 |
| Mtx2 | -0.2432752 | 0.01865065 |
| Grsf1 | -0.2469377 | 0.01031901 |
| Mrpl38 | -0.2649995 | 0.03542831 |
| Ndufa11 | -0.2679445 | 0.02862335 |
| Tmem205 | -0.2712026 | 0.03921005 |
| Atp5g3 | -0.2723823 | 0.00277479 |
| Ndufb8 | -0.2729551 | 0.01712048 |
| Mrpl47 | -0.2822727 | 0.02671559 |
| Ndufv1 | -0.2837318 | 0.00065105 |
| Fis1 | -0.2837516 | 0.00735915 |
| Fahd1 | -0.2902542 | 0.04031112 |
| Mrpl44 | -0.2954818 | 0.01687555 |
| Letmd1 | -0.2955642 | 0.00323209 |
| Carkd | -0.3041876 | 0.02927492 |
| Letm1 | -0.3043026 | 0.00939971 |
| Fastk | -0.3083121 | 0.00511508 |
| Supv3l1 | -0.3118373 | 0.02793399 |
| Slc25a16 | -0.31646 | 0.0078402 |
| Hint2 | -0.3200087 | 0.00021761 |
| Polrmt | -0.3203616 | 0.0105124 |
| Lypla1 | -0.3220057 | 5.40E-06 |
| Coa3 | -0.3249265 | 0.03147849 |
| Slc25a23 | -0.3274361 | 0.00125611 |
| Bdh1 | -0.3299838 | 0.01220442 |
| Mrpl4 | -0.343744 | 0.00529801 |
| Mdh2 | -0.3627366 | 0.00693381 |
| Acads | -0.3638785 | 0.02962767 |
| Slc25a39 | -0.3668757 | 0.02059974 |
| L2hgdh | -0.3704586 | 0.02984111 |
| Malsu1 | -0.3720708 | 0.00636471 |
| Pxmp2 | -0.3813803 | 8.17E-07 |
| Mmadhc | -0.3822636 | 0.00297037 |
| Dlat | -0.3841671 | 0.00299836 |
| Gfm1 | -0.3880354 | 1.69E-09 |
| Mrps11 | -0.3942014 | 0.0067309 |
| Macrod1 | -0.3985363 | 0.04617768 |
| Mpv17l2 | -0.4009924 | 0.0124062 |
| Fxn | -0.4049257 | 0.03841539 |
| Nif3l1 | -0.4051532 | 0.01256608 |
| Tmem223 | -0.4105728 | 0.00630907 |
| Mtif2 | -0.4111228 | 0.00044023 |
| Ptpmt1 | -0.4148737 | 1.20E-05 |
| Ndufc2 | -0.4184936 | 6.17E-06 |
| Ndufv3 | -0.4232011 | 2.59E-05 |
| Acat1 | -0.4240027 | 9.12E-08 |
| Mrpl10 | -0.4242601 | 0.00395896 |
| Abcb6 | -0.4303769 | 3.63E-06 |
| Lyrm2 | -0.4318487 | 0.00121978 |
| Adhfe1 | -0.4333372 | 0.00031262 |
| Ndufa12 | -0.4346558 | 0.02592905 |
| Myg1 | -0.4401588 | 0.00224875 |
| Ivd | -0.4420473 | 0.00056564 |
| Ndufaf5 | -0.4427027 | 0.0455906 |
| Mrpl20 | -0.4460129 | 0.0006817 |
| Acss3 | -0.449925 | 0.00011701 |
| Dhrs4 | -0.4499545 | 0.01391464 |
| Sdhc | -0.4582875 | 0.0004677 |
| Tmem70 | -0.460255 | 0.0029696 |
| Dnajc4 | -0.4602628 | 0.01063661 |
| Mrpl22 | -0.4609461 | 0.01311941 |
| Ndufb7 | -0.4619337 | 0.00422178 |
| Trap1 | -0.4669218 | 0.00262955 |
| Glrx5 | -0.4691857 | 0.01915916 |
| Auh | -0.4754072 | 0.01003024 |
| Mrps24 | -0.4782082 | 0.0042929 |
| Acadm | -0.478754 | 0.03784108 |
| Coasy | -0.4800275 | 8.98E-06 |
| Gcdh | -0.483233 | 0.0109049 |
| Sdr39u1 | -0.4883809 | 0.02414158 |
| Mrpl50 | -0.4933644 | 3.91E-06 |
| Mrpl50 | -0.4933644 | 3.91E-06 |
| Ptcd1 | -0.5049288 | 0.00121655 |
| Nme3 | -0.5070594 | 0.00860605 |
| Acp6 | -0.5099672 | 1.74E-09 |
| Nme6 | -0.5108668 | 0.00064877 |
| Mrpl37 | -0.5117756 | 1.08E-08 |
| Ndufs7 | -0.519739 | 0.0001253 |
| Gstz1 | -0.5257427 | 0.00316327 |
| Bckdha | -0.5287955 | 0.00017481 |
| Pars2 | -0.5305285 | 0.03787732 |
| Samm50 | -0.53202 | 9.85E-08 |
| Mfn2 | -0.5326323 | 1.13E-05 |
| Mrpl14 | -0.5343642 | 0.00674007 |
| Acadvl | -0.5375863 | 9.70E-13 |
| Acaa2 | -0.5403837 | 0.0326496 |
| Scp2 | -0.5404918 | 0.00056296 |
| Slc30a6 | -0.5415731 | 0.0002344 |
| Mpc2 | -0.548578 | 1.57E-12 |
| Gadd45gip1 | -0.549838 | 1.00E-08 |
| Acadl | -0.5575273 | 5.76E-28 |
| Tars2 | -0.5607442 | 3.93E-05 |
| Abcb8 | -0.5610737 | 0.01652062 |
| Nit1 | -0.5649794 | 0.00130476 |
| Pink1 | -0.5653548 | 0.00017346 |
| Hsd17b10 | -0.5717534 | 0.00393355 |
| Pck2 | -0.5758751 | 0.02451788 |
| Cox19 | -0.5807163 | 2.75E-09 |
| Nit2 | -0.594584 | 7.63E-06 |
| Bcat2 | -0.6085481 | 0.00885504 |
| Mrps16 | -0.6108566 | 4.73E-08 |
| Ecsit | -0.6127252 | 4.71E-06 |
| Echdc2 | -0.6145703 | 1.90E-05 |
| Chchd10 | -0.6191291 | 0.00015244 |
| Ak2 | -0.6199835 | 0.00374353 |
| Mdh1 | -0.623033 | 0.00450695 |
| Fundc1 | -0.6303506 | 0.00017338 |
| Hint3 | -0.6349029 | 1.69E-05 |
| Ndufb10 | -0.6361326 | 3.93E-06 |
| Eefsec | -0.6447232 | 6.77E-06 |
| Prodh2 | -0.6479978 | 0.00042709 |
| Adck2 | -0.6674666 | 1.21E-05 |
| Prosc | -0.6682412 | 1.47E-05 |
| Mccc1 | -0.6723536 | 4.03E-14 |
| Hadha | -0.6794245 | 7.11E-14 |
| Mrpl2 | -0.6956192 | 1.07E-05 |
| Rmnd1 | -0.7008075 | 0.00018551 |
| Them4 | -0.7031773 | 0.03118781 |
| Mtfp1 | -0.7115396 | 2.51E-05 |
| Cbr4 | -0.7245798 | 9.77E-08 |
| Eci2 | -0.7261805 | 3.04E-09 |
| Gfm2 | -0.7265473 | 0.00029192 |
| Slc25a11 | -0.7335383 | 4.36E-13 |
| Grhpr | -0.7526151 | 6.22E-11 |
| Ndufa8 | -0.7537544 | 1.18E-05 |
| Polg2 | -0.7643595 | 0.00041374 |
| Aldh1l1 | -0.7647636 | 0.00056888 |
| Qrsl1 | -0.7722149 | 0.00053519 |
| Dcxr | -0.7811623 | 0.00709081 |
| Aco2 | -0.7818258 | 2.52E-10 |
| Pdhb | -0.8074774 | 0.00085614 |
| Mtfmt | -0.8092644 | 2.42E-07 |
| Mecr | -0.8117205 | 0.0010887 |
| Coq9 | -0.8222676 | 2.05E-14 |
| Tcirg1 | -0.830292 | 1.76E-15 |
| Rars2 | -0.830529 | 2.35E-08 |
| Aldh1l2 | -0.8393485 | 4.32E-05 |
| Cpox | -0.8436946 | 2.90E-10 |
| Abcd1 | -0.8539585 | 1.97E-07 |
| Nudt6 | -0.8580476 | 6.07E-05 |
| Atpaf1 | -0.8612466 | 6.57E-06 |
| Tst | -0.8823154 | 0.00040185 |
| Gtpbp3 | -0.8839395 | 3.94E-05 |
| Bphl | -0.8845051 | 1.17E-09 |
| Sirt5 | -0.8848969 | 1.36E-10 |
| Slc25a42 | -0.8929281 | 3.90E-07 |
| Acaa1a | -0.9070647 | 0.00014124 |
| Rpusd3 | -0.9183341 | 8.31E-06 |
| Acad9 | -0.9331842 | 1.74E-07 |
| Hmbs | -0.9390045 | 5.53E-07 |
| Oxsm | -0.9397542 | 1.17E-08 |
| Cryz | -0.940312 | 1.79E-09 |
| Dcakd | -0.9429442 | 0.00103637 |
| Coa4 | -0.958002 | 2.92E-07 |
| Tmem14c | -0.9611863 | 6.32E-17 |
| Etfb | -0.9908264 | 1.51E-07 |
| Fech | -1.0015691 | 2.28E-10 |
| Tmem126a | -1.0050018 | 1.17E-08 |
| Chdh | -1.0103552 | 5.40E-05 |
| Hadhb | -1.0138514 | 1.89E-19 |
| Aldh5a1 | -1.0364694 | 2.54E-07 |
| Pdk2 | -1.0809978 | 0.00904374 |
| Nlrx1 | -1.1011211 | 1.29E-09 |
| Acss1 | -1.1102722 | 0.00164756 |
| Acad11 | -1.1113067 | 1.20E-07 |
| Alas1 | -1.1123913 | 2.21E-36 |
| Slc25a40 | -1.1171363 | 0.00378491 |
| Glyctk | -1.1320903 | 3.65E-50 |
| Fdxr | -1.149589 | 2.13E-07 |
| Abcd3 | -1.161997 | 6.05E-70 |
| Nudt13 | -1.2359139 | 9.06E-05 |
| Sfxn4 | -1.2476392 | 0.0124062 |
| Slc25a20 | -1.2502562 | 4.09E-13 |
| Acsf2 | -1.3066387 | 1.04E-16 |
| Mutyh | -1.3119098 | 0.00010075 |
| Sirt3 | -1.3144669 | 3.49E-09 |
| Fam210b | -1.424658 | 9.10E-08 |
| Cpt2 | -1.5633422 | 5.26E-32 |
| Tysnd1 | -1.5753281 | 2.12E-14 |
| Plgrkt | -1.6913343 | 4.10E-06 |
| Ech1 | -1.7409329 | 2.23E-20 |
| Ephx2 | -2.7502406 | 3.00E-08 |
| Abcd2 | -2.9582363 | 3.62E-08 |
| Hdhd3 | -3.039709 | 1.15E-14 |

| **Table 3: b) Nuclear genes encoding mitochondrial proteins that were significantly differentially expressed (adjusted P-value<0.05) following sepsis in the rat liver compared to control (no sepsis + propofol) in Propofol background.** |  |  |
| --- | --- | --- |
| **Gene** | **log2 fold change** | **adjusted P-value** |
| Slc25a29 | 2.10158173 | 2.18E-16 |
| Pptc7 | 1.87073646 | 5.12E-16 |
| Aifm3 | 1.628176 | 0.01092001 |
| Coq10b | 1.62644943 | 2.00E-13 |
| Pdp1 | 1.02800656 | 6.56E-08 |
| Rfk | 0.96681931 | 1.22E-09 |
| Gls | 0.9401475 | 0.00023465 |
| Hk1 | 0.91322782 | 0.00966919 |
| Lamc1 | 0.78188112 | 2.34E-07 |
| Txndc12 | 0.76760889 | 9.96E-11 |
| Rab32 | 0.54819439 | 0.00385037 |
| Adck4 | 0.53950154 | 2.05E-07 |
| Phyh | 0.49369891 | 4.00E-07 |
| Slc25a19 | 0.47593731 | 0.00153501 |
| Bad | 0.46913509 | 0.0044176 |
| Bcl2l2 | 0.4451964 | 1.13E-05 |
| Bnip3l | 0.41926347 | 0.0010539 |
| Dnlz | 0.39273257 | 0.00256171 |
| Oxnad1 | 0.38992276 | 6.22E-08 |
| Pdha1 | 0.34851202 | 0.00582989 |
| Htra2 | 0.34449511 | 0.00849196 |
| Cisd2 | 0.31531538 | 0.00122417 |
| Rab24 | 0.27267662 | 0.00506198 |
| Bax | 0.26578114 | 0.01778513 |
| Ndufs2 | 0.23755479 | 0.00990729 |
| Rhot2 | 0.23305399 | 0.03354652 |
| Opa1 | 0.2136203 | 0.0143282 |
| Nrd1 | 0.17712872 | 0.01935405 |
| Guk1 | 0.16184577 | 0.03565562 |
| Selo | -0.1659639 | 0.036129 |
| Sdhc | -0.1701833 | 0.04739773 |
| Pccb | -0.1758504 | 0.01276541 |
| Slc25a44 | -0.1851994 | 0.0477845 |
| Abcb6 | -0.1906111 | 0.03860575 |
| Gcdh | -0.2057281 | 0.02415614 |
| Ifi27 | -0.2114599 | 0.01478551 |
| Oxa1l | -0.2314716 | 0.03994108 |
| Fkbp8 | -0.2366613 | 0.00233462 |
| Timm8a1 | -0.2416572 | 0.04871024 |
| Slc25a16 | -0.2434982 | 0.00638762 |
| Mrpl10 | -0.2490589 | 0.04136156 |
| Pxmp2 | -0.250981 | 0.00031175 |
| Mrps30 | -0.2511908 | 0.0373945 |
| Supv3l1 | -0.2566647 | 0.02186083 |
| Letmd1 | -0.2591399 | 0.01606952 |
| Acads | -0.2652268 | 0.0132276 |
| Mrpl47 | -0.2683033 | 0.04655452 |
| Agxt2 | -0.2684195 | 6.92E-05 |
| Mfn2 | -0.2743487 | 0.02051287 |
| Tomm70a | -0.2745607 | 0.02533353 |
| Mdh1 | -0.2767111 | 0.00978101 |
| Acp6 | -0.2851888 | 0.01742352 |
| Mrpl37 | -0.2854488 | 0.01365224 |
| Mrps16 | -0.2892381 | 0.02304177 |
| Ecsit | -0.2893745 | 0.03628768 |
| Pdhx | -0.2919638 | 0.01165411 |
| Lypla1 | -0.2928086 | 0.00209063 |
| Tmem205 | -0.2937949 | 0.00060336 |
| Prosc | -0.2964422 | 0.04459275 |
| Bckdha | -0.3059184 | 0.00970379 |
| Pus1 | -0.3085187 | 0.0477845 |
| Gars | -0.312452 | 0.01187332 |
| Acadvl | -0.3165443 | 0.00024865 |
| Acadm | -0.317353 | 0.000653 |
| Tmem223 | -0.3179916 | 0.02984507 |
| Lap3 | -0.3214463 | 0.00034192 |
| Slc25a39 | -0.3224816 | 3.38E-05 |
| Dcxr | -0.3231474 | 0.01859588 |
| Ndufb10 | -0.3388168 | 0.01422212 |
| Mccc1 | -0.3499965 | 0.01784855 |
| Hsd17b10 | -0.3552006 | 0.00048855 |
| Tk2 | -0.3609624 | 0.01262604 |
| Acat1 | -0.3696975 | 0.00146772 |
| Nif3l1 | -0.369905 | 0.01749757 |
| Gstz1 | -0.3744804 | 0.00011781 |
| Hmbs | -0.3776096 | 0.01604705 |
| Tars2 | -0.3784946 | 0.00383596 |
| Acad9 | -0.3846377 | 0.00227635 |
| Adck2 | -0.3851925 | 0.00414517 |
| Dbi | -0.3873837 | 0.00121432 |
| Grsf1 | -0.3889488 | 2.00E-06 |
| Trap1 | -0.3945962 | 0.00044077 |
| Mtfp1 | -0.4013229 | 0.00229711 |
| Hars2 | -0.4016227 | 0.00023139 |
| Tmem126a | -0.4043387 | 0.00398363 |
| Mrpl2 | -0.4052297 | 0.0154135 |
| Ndufv3 | -0.4065809 | 0.00416661 |
| Aco2 | -0.4195152 | 0.00220535 |
| Mrpl50 | -0.4200664 | 0.00102155 |
| Mrpl50 | -0.4200664 | 0.00102155 |
| Ndufa8 | -0.4230661 | 0.00016354 |
| Mrpl44 | -0.4246997 | 0.00076916 |
| Coasy | -0.430678 | 0.000355 |
| Gtpbp3 | -0.4309216 | 0.00970137 |
| Adhfe1 | -0.4337253 | 3.67E-06 |
| Scp2 | -0.4342013 | 3.33E-07 |
| Ptcd1 | -0.4355695 | 0.00286221 |
| Cryz | -0.4374365 | 0.03100366 |
| Myg1 | -0.4474584 | 0.00221095 |
| Dhrs4 | -0.449185 | 0.00041483 |
| Eci2 | -0.4493529 | 6.49E-08 |
| Oxsm | -0.4543936 | 0.00585717 |
| Acad11 | -0.4556586 | 0.00178939 |
| Rmnd1 | -0.462097 | 0.0002651 |
| Bckdk | -0.4629628 | 4.64E-05 |
| Nthl1 | -0.4787863 | 0.01810644 |
| Qrsl1 | -0.4794546 | 0.03001307 |
| Atpaf1 | -0.4809284 | 0.00015881 |
| Mpc2 | -0.4825256 | 4.64E-05 |
| Nit2 | -0.4860613 | 2.20E-07 |
| Acadl | -0.4867136 | 2.09E-07 |
| Polrmt | -0.4909314 | 9.83E-07 |
| Fundc1 | -0.4913228 | 0.00747227 |
| Pdhb | -0.4947763 | 0.01256526 |
| Gsr | -0.4995489 | 4.52E-05 |
| Grhpr | -0.5032477 | 5.42E-09 |
| Slc25a32 | -0.5118311 | 7.01E-07 |
| Etfb | -0.5128939 | 5.79E-08 |
| Cbr4 | -0.5147389 | 0.04132964 |
| Prodh2 | -0.5152932 | 2.41E-05 |
| Auh | -0.5172386 | 0.00014024 |
| Ndufaf4 | -0.5181646 | 0.00077248 |
| Malsu1 | -0.519073 | 0.00317615 |
| Aldh1l2 | -0.5192785 | 0.00432204 |
| Nme3 | -0.5212814 | 0.00356712 |
| Rars2 | -0.5236834 | 0.00378948 |
| Bphl | -0.5254656 | 0.00930134 |
| Echdc2 | -0.5365067 | 3.26E-05 |
| Cyp27a1 | -0.5425216 | 2.98E-07 |
| Hadha | -0.5443325 | 1.85E-13 |
| Aldh1l1 | -0.5474294 | 0.00137348 |
| Coq9 | -0.5518562 | 0.00010436 |
| Bcs1l | -0.5523162 | 0.00067221 |
| Nme6 | -0.5537153 | 0.00769409 |
| Slc25a11 | -0.5563512 | 1.42E-05 |
| Fech | -0.5575478 | 2.12E-06 |
| Chdh | -0.5648835 | 0.01113786 |
| Slc25a42 | -0.566436 | 0.00019348 |
| Acsf2 | -0.5790386 | 2.97E-07 |
| Chchd10 | -0.5919016 | 4.54E-08 |
| Slc25a30 | -0.5948165 | 0.02591565 |
| Fdxr | -0.6017888 | 0.03400711 |
| Mrpl14 | -0.6332037 | 0.00867948 |
| Slc25a37 | -0.6380898 | 0.00454636 |
| Coa4 | -0.6399493 | 0.00039141 |
| Nit1 | -0.6638827 | 1.33E-08 |
| Slc25a20 | -0.6659065 | 1.54E-08 |
| Mtfmt | -0.6852399 | 3.52E-06 |
| Hint3 | -0.6893341 | 0.00016195 |
| Bdh1 | -0.6920139 | 2.29E-09 |
| Tst | -0.6958939 | 1.13E-11 |
| Hadhb | -0.6975714 | 1.08E-11 |
| Cpox | -0.7041431 | 2.55E-12 |
| Eefsec | -0.7059383 | 7.78E-08 |
| Slc25a33 | -0.7145149 | 6.91E-05 |
| Tcirg1 | -0.7186304 | 3.08E-05 |
| Dcakd | -0.7224612 | 0.00391512 |
| Nudt13 | -0.7263412 | 0.04391957 |
| Bcat2 | -0.7316498 | 0.00231609 |
| Acot2 | -0.7353091 | 0.04256993 |
| Aldh5a1 | -0.7551122 | 2.83E-06 |
| Fahd1 | -0.759142 | 1.23E-07 |
| Rpusd3 | -0.7625893 | 0.00101776 |
| Nlrx1 | -0.7800233 | 0.00039119 |
| Abcd1 | -0.7861571 | 1.64E-05 |
| Nudt6 | -0.8020174 | 0.0015023 |
| Sirt3 | -0.8261121 | 5.18E-12 |
| Tmem14c | -0.8270652 | 1.02E-07 |
| Glyctk | -0.8525429 | 4.58E-05 |
| Tysnd1 | -0.9077794 | 2.24E-09 |
| Sirt5 | -0.9365071 | 9.31E-09 |
| Fam210b | -0.940831 | 4.57E-06 |
| Abcd3 | -0.9462524 | 5.68E-11 |
| Acss1 | -0.9666039 | 0.04612208 |
| Acaa1a | -1.0046839 | 4.98E-05 |
| Polg2 | -1.0834407 | 5.97E-08 |
| Acaa2 | -1.135318 | 6.97E-13 |
| Ech1 | -1.1887189 | 2.79E-10 |
| Mutyh | -1.1997047 | 0.00054083 |
| Plgrkt | -1.2921067 | 4.23E-05 |
| Pdk2 | -1.3882242 | 7.54E-13 |
| Cpt2 | -1.4935521 | 6.93E-19 |
| Abcd2 | -1.7714374 | 0.00012683 |
| Alas1 | -1.9041492 | 5.08E-18 |
| Hdhd3 | -2.6715179 | 8.61E-31 |
