# Supplementary material for: Anesthesia-Sepsis-Associated Alterations in Liver Gene Expression Profiles and Mitochondrial Oxidative Phosphorylation Complexes
Source: Front Med (Lausanne). 2020 Dec 18;7:581082. doi: 10.3389/fmed.2020.581082 (PMC7775734; doi:10.3389/fmed.2020.581082)
Supplement: Supplementary file 4 [file Data_Sheet_1.docx]

**Supplementary material data sheet 1 (**Immuno blots)

Data sheet 1A-Immuno blots (Isoflurane and Sepsis group)


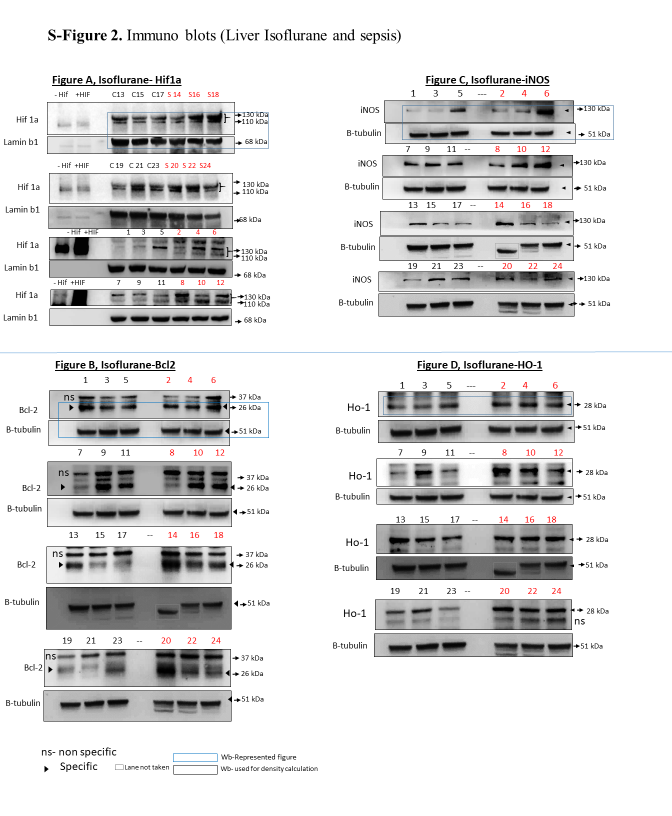


**Data 1A.**Immuno blots (Liver Isoflurane and sepsis) used for calculating protein expression of HIF1a, Bcl2, iNOS and HO-1, showed in main text **figures (1)** A, B, C and D.

Data sheet 1B-Immuno blots (Propofol and Sepsis group)


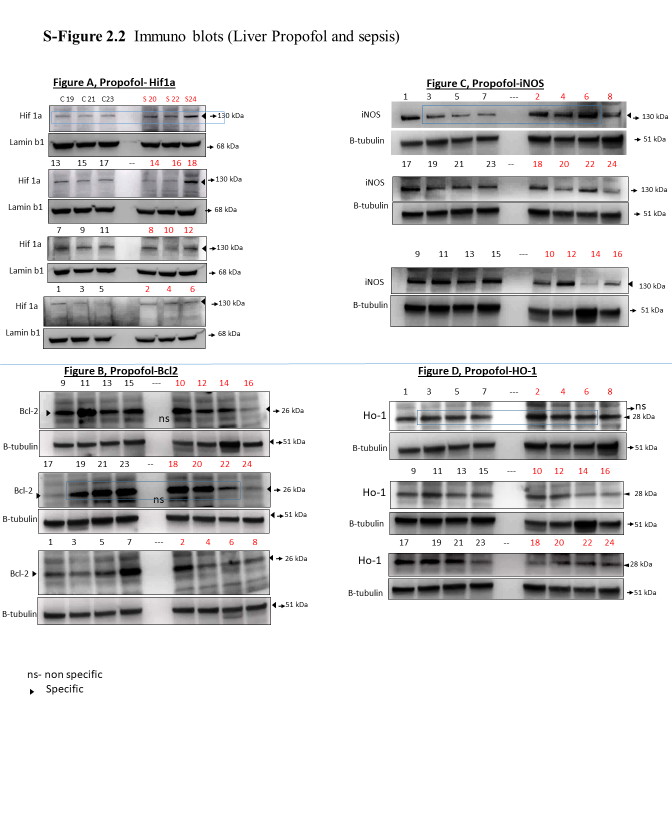


**Data 1B.** Immuno blots (Propofol and sepsis group) used for calculating protein expression of HIF1a, Bcl2, iNOS and HO-1, showed in main text figures (1) A, B, C and D.

**Data sheet 1C.** S-nitrosylation Ponceau-s staining blots


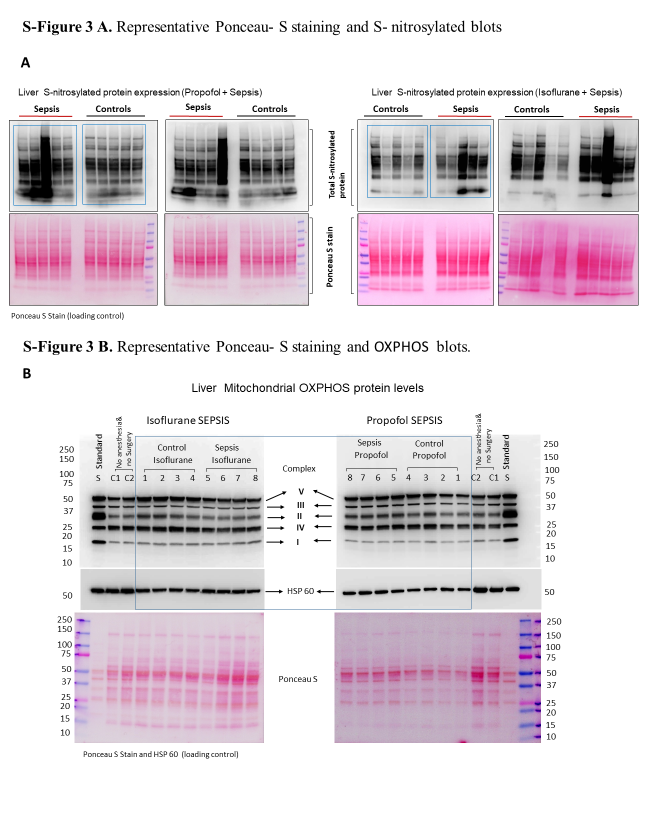


**Data sheet 1D**. Immuno blots displaying Complexes I-V Protein expression levels and HSP 60 (loading

control) and Ponceau-s staining.


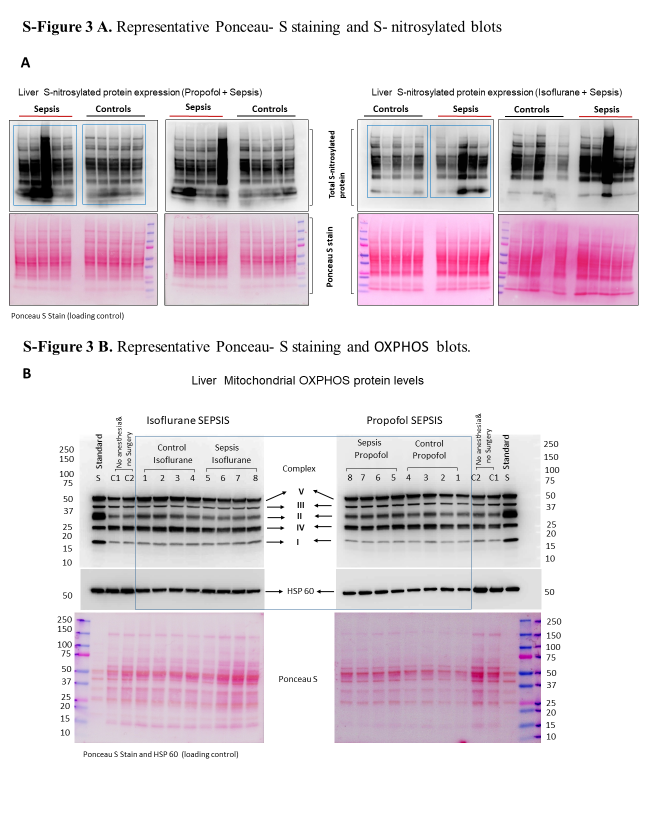


**Data**. (**1C**). Immuno blots (S- nitrosylated) and Ponceau- S staining blots (loading control) used for calculating total S- nitrosylated proteins showed in main text figure 2,D. **(1D)** Immuno blots (OXPHOS) and loading control (HSP 60) and additional loading control Ponceau- S staining blots used for calculating Mitochondrial Oxidative Phosphorylation (OXPHOS) Complexes I-V Protein expression levels showed in main text figure 3,B.

Indicates represented blot used in main text
