# Supplementary material for: Anesthesia-Sepsis-Associated Alterations in Liver Gene Expression Profiles and Mitochondrial Oxidative Phosphorylation Complexes
Source: Front Med (Lausanne). 2020 Dec 18;7:581082. doi: 10.3389/fmed.2020.581082 (PMC7775734; doi:10.3389/fmed.2020.581082)
Supplement: Supplementary file 5 [file Data_Sheet_2.docx]

**Supplemental Digital Content: data sheet 2 (Methodology).** Methodology related to HIF-1 and other proteins immunoblotting, quantitative PCR-Primers list and Additional information references.

**Methodology**

For detailed, Tissue process, protein preparation, HIF-1 and other protein immunoblotting standardization, please see our research group previously published Supplemental Digital Content (https://link.springer.com/article/10.1186/s12868-019-0514-8#additional-information) with Brain subcellular energetics data; Thiele et al., BMC Neurosci 2019, 20, 34,https://doi.org/10.1186/s12868-019-0514-8.

**Methodology**

HIF-1 is a highly labile protein and is degraded by prolyl hydroxylase within minutes of exposure to atmospheric levels of oxygen (Huang LE, et al 1996). Thus, tissue handling can be a challenge.

**Tissue Preparation**

Immediately prior to analysis, the extracted tissue was removed from storage at -80C and individually finely ground in liquid N_2_ with a pestle and mortar. During this process, we constantly poured liquid nitrogen on the sample to ensure that it remained below -80C. Ten to fifty milligrams of liver tissue powder from each sample were used to measure Gene, protein expression, and oxidative stress markers.

Specific Methodology (all proteins):

50 mg of liver tissue powder was suspended in 500 ul of ice cold PBS containing a commercially-prepared protease and phosphatase inhibitor cocktail (Halt^TM^, Thermo -Fisher #78443, Waltham, MA). Samples were gently mixed, centrifuged at 10,000 rpm for 5 minutes at 4^0^C, and the pellet was collected and used either for nuclear protein extraction or for total protein lysis preparation (this is a washing step to remove blood or other tissue contaminants). Nuclear proteins were extracted using commercially available mammalian cell and tissue nuclear extraction kit (abcam ab113474, Cambridge, MA) by following the manufacturer’s protocol with the exception of adding 100 uM CoCl_2_ to the extraction buffer (we used RIPA for this stage of the nuclear extraction because its contents were more clearly described) in order to stabilize prolyl hydroxylase and thereby preventing HIF-1 degradation in the presence of atmospheric oxygen (Srinivasan S et al 2011).

For total protein extraction, 250 uL of ice cold lysis buffer (RIPA) containing a protease and phosphatase inhibitor cocktail (Halt^TM^) was added to the pellet, which was subsequently sonicated on ice (3 cycles of 10 pulses, power 5, Fisher Scientific Sonic Dismembrator Model F60, Pittsburgh, PA). Protein lysates were centrifuged at 13,000 rpm for 20 minutes at 4^0^C.

Protein concentrations were determined using BCA kit (Thermo Scientific). 10-30 μg of total protein (50 ug for HIF-1α) was heat-denatured at 95C, loaded onto 4 to 20% Tris-Glycine polyacrylamide gradient gels (Bio-Rad, Hercules, CA), and electrophoresed. Proteins were transferred to PVDF membrane (Millipore, Darmstadt, Germany), blocked at room temperature for 1 h in SuperBlock (PBS) Blocking Buffer (Thermo-Fisher # 37515) and incubated at 4°C overnight with primary antibodies; anti-HIF-1α (1:2000, rabbit –mAb # 14179, Cell Signaling [Danvers, MA]; note: for HIF-1α, overnight incubation with primary antibodies was repeated after washing three times with PBST), anti-HO-1 (1:3000, mouse- mAb # NBP1-97507, NovusBio [Littleton, CO]), anti-bcl2 (1:5000, mouse- mAb # NB100-78543, NovusBio), anti-iNOS (1:1500, rabbit-mAb # 13120, Cell signaling). For loading controls, anti-β-tubulin (1:2000, mouse-mAb # NB 120-7792, NovusBio) and anti-Lamin B1 (1:2000, rabbit-mAb # 13435, Cell Signaling) were used.

After primary antibody incubation, membranes were washed three times in PBST for 10 minutes. Membranes were then incubated with horseradish peroxidase–conjugated secondary antibodies (1:1000-15,000, depending on the primary antibody used, Santa Cruz Biotechnology [Dallas, TX] and Cell Signaling], for 1h at room temperature. Immunoreactivity was detected using enhanced chemiluminescence substrate (Super Signal West Femto; Thermo Scientific). Images were captured using GBOX (Chemi XR5; Syngene), and gels were analyzed densitometrically using the computerized image analysis software (Gene Tools from Syngene). Target protein bands were normalized to loading controls β-tubulin or Lamin B1.

|  | **HIF-1** | **Bcl-2** | **iNOS** | **HO-1** |
| --- | --- | --- | --- | --- |
| Location | Nuclear | Total Protein Lysate | Total Protein Lysate | Total Protein Lysate |
| Gel | 4-20% TGX 50ul capacity | 4-20% TGX 20-30 ul capacity | 4-20% TGX 20-30 ul capacity | 4-20% TGX 20-30 ul capacity |
| Protein loaded | 50 ug | 25 ug | 25 ug | 25 ug |
| Primary Antibody | rabbit –mAb # 14179  Cell signaling | mouse- mAb # NB100-78543  NovusBio | rabbit-mAb # 13120  Cell signaling | mouse- mAb # NBP1-97507  NovusBio |
| Primary Dilution | 1:2000 | 1:5000 | 1:1500 | 1:3000 |
| Secondary antibody | Rabbit | Mouse | Rabbit | Mouse |
| Secondary dilution | 1:15000 Santa Cruz | 1:1000 Cell Signaling | 1:10000 Cell Signaling | 1:15000 Cell Signaling |
| Loading Control | Lamin B1 | β−tubulin | β−tubulin | β−tubulin |

**Quantitative PCR** Primers used for Mitochondrial DNA levels quantification and RNASeq data validation.

All primers were purchased from Sigma and Invitrogen, USA.

| **Gene** | **Forward (5'-3')** | **Reverse (5'-3')** |
| --- | --- | --- |
| *Ndufv2* | GACTACTACGAGGATCTGAC | GGTTCAGTCAAAGAAGTAAGG |
| *Ndufs1* | GATTTGCCTATGATGGACTG | TGCCTTCAAAACTCTTGTAAC |
| *Sdhd* | TTGTCAGTGTTTTGCTCTTG | CATGAACGTAGTCAGTAACC |
| *Sdhb* | CAACAGTATCTGCAATCCATC | GTGAATTCATCTCTGGAGTC |
| *Uqcrh* | CAAAGAGGAAGAAGAGGAAG | AAGAGCTCCTCTGTACAATC |
| *Uqcrb* | CAGCTCTCTACTCTTAGGTC | CTGCATTATAATACCACTTCCG |
| *Cox8a* | ATATCACCATTGGGCTCAC | AAAGGATGAGGGAAGACG |
| *Cox7a2l* | AGCTACAGAGATTCTTCCAG | CACAAAGTAAACCAGTCCTC |
| *Atp5c1* | CTGCTCTGGATTAATGAGAATC | CTAACAAGGACAGCAATCAG |
| *Atp5j* | TGGACAGAGAGCTTTTTAAG | GGATCCTCAAAATTGAAGGTAG |
| *Atp6ap1l* | ATTTAAGAATCAGACGTGGC | CTGGACAGCTTGATGTAATTG |
| *Tuba1a* | ATTATGAGGAGGTTGGTGTG | TGTTGGACCAGAATAAACATG |
| *Mt_Nd1* | GCAGGACCATTCGCCCTATT | GGGGTAGGATGCTCGGATTC |
| *Mt_Nd6* | CGCAAACAATGACCACCCAG | CCCGGAGACTTGAGGGTCTA |

**Additional Supplemental information References**

1. Huang LE, Arany Z, Livingston DM, Bunn HF. Activation of hypoxia-inducible transcription factor depends primarily upon redox-sensitive stabilization of its alpha subunit. *J Biol Chem.* 1996;271(50):32253-32259.
2. Srinivasan S, Dunn JF. Stabilization of hypoxia-inducible factor-1alpha in buffer containing cobalt chloride for Western blot analysis. *Anal Biochem.* 2011;416(1):120-122.
3. Thiele, R. H., H. P. Osuru, U. Paila, K. Ikeda, and Z. Zuo. 2019. 'Impact of inflammation on brain subcellular energetics in anesthetized rats', BMC Neurosci, 20: 34.
4. Schisterman, E. F., and A. Vexler. 2008. 'To pool or not to pool, from whether to when: applications of pooling to biospecimens subject to a limit of detection', Paediatr Perinat Epidemiol, 22: 486-96.
5. Urena-Peralta, J. R., S. Alfonso-Loeches, C. M. Cuesta-Diaz, F. Garcia-Garcia, and C. Guerri. 2018. 'Deep sequencing and miRNA profiles in alcohol-induced neuroinflammation and the TLR4 response in mice cerebral cortex', Sci Rep, 8: 15913.
6. Goodall, E. F., V. Leach, C. Wang, J. Cooper-Knock, P. R. Heath, D. Baker, D. R. Drew, M. J. Saffrey, J. E. Simpson, I. A. Romero, and S. B. Wharton. 2019. 'Age-Associated mRNA and miRNA Expression Changes in the Blood-Brain Barrier', Int J Mol Sci, 20.
