# Supplementary material for: Anesthesia-Sepsis-Associated Alterations in Liver Gene Expression Profiles and Mitochondrial Oxidative Phosphorylation Complexes
Source: Front Med (Lausanne). 2020 Dec 18;7:581082. doi: 10.3389/fmed.2020.581082 (PMC7775734; doi:10.3389/fmed.2020.581082)
Supplement: Supplementary file 6 [file Image_1.pdf]

Supplementary Figure 1, Volcano plot of differentially expressed genes in sepsis,

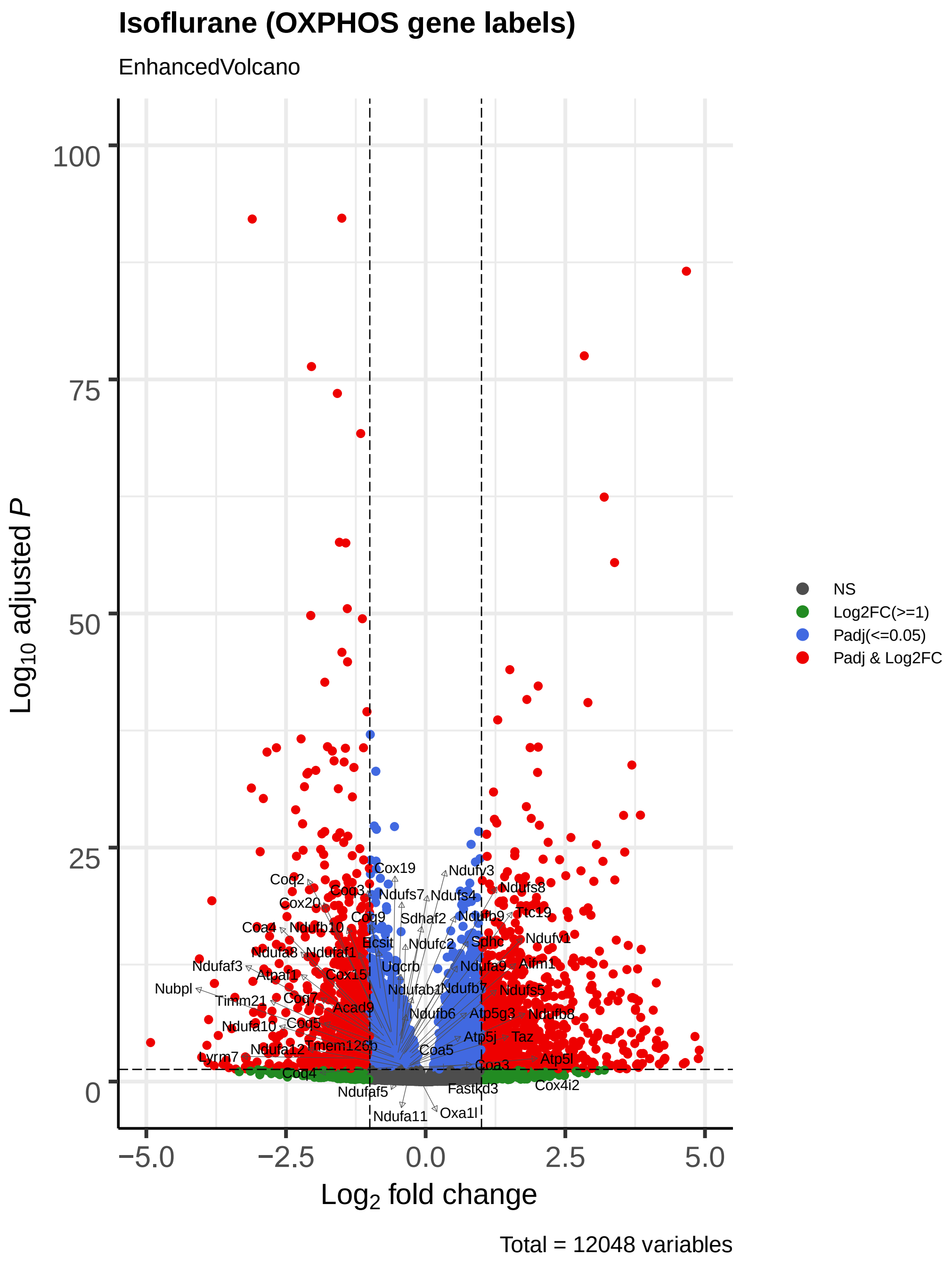

Figure 1. (A) Volcano plot of differentially expressed genes in sepsis with an Isoflurane background. Genes related to OXPHOS that were significant (adjusted P-value<0.05 and log2 fold change>1) are labelled in the plot.

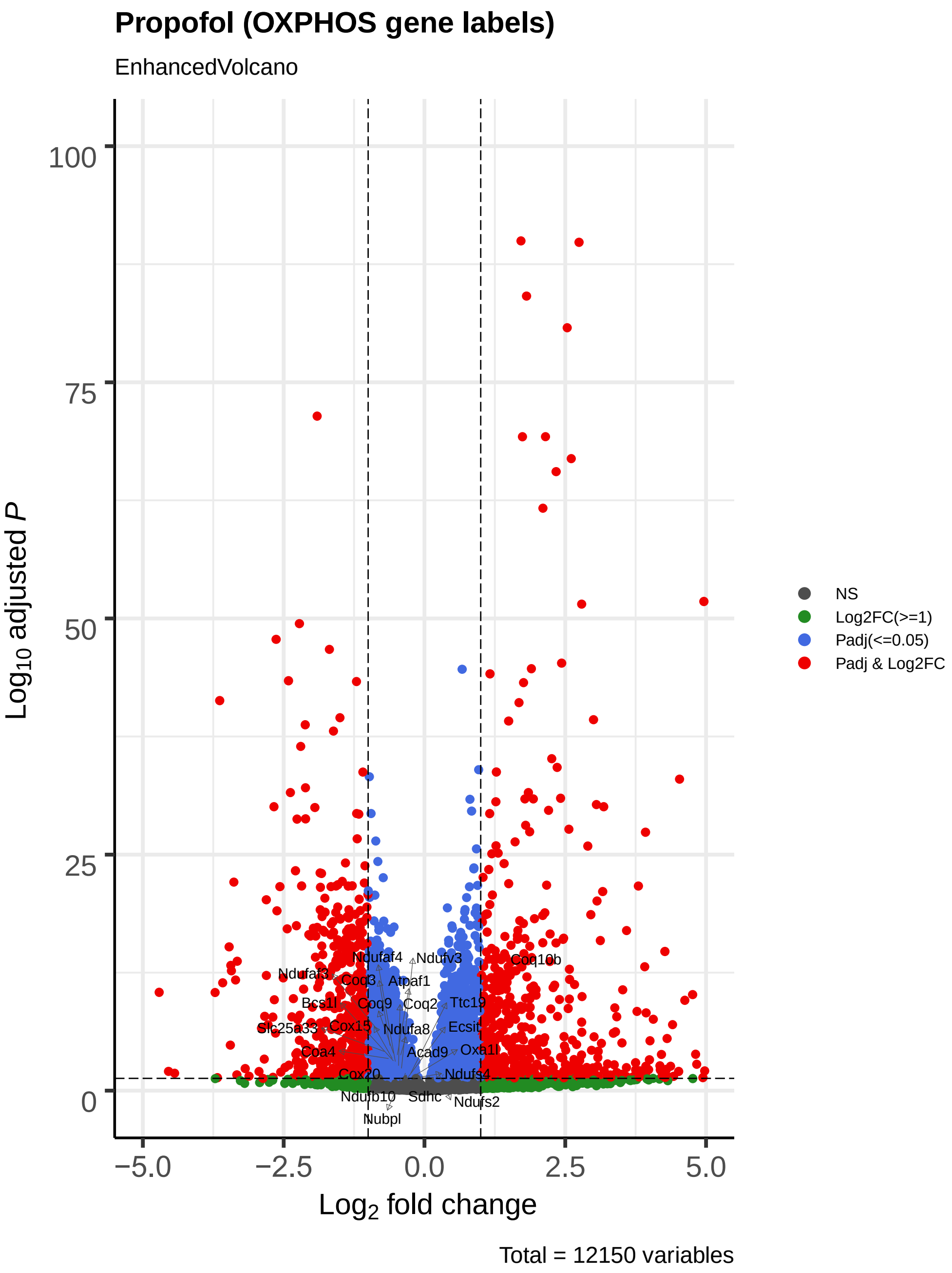

<u>Figure 1</u>. (B) Volcano plot of differentially expressed genes in sepsis with a Propofol background. Genes related to OXPHOS that were significant (adjusted P-value<0.05 and log2 fold change>1) are labelled in the plot.
