# Supplementary material for: Anesthesia-Sepsis-Associated Alterations in Liver Gene Expression Profiles and Mitochondrial Oxidative Phosphorylation Complexes
Source: Front Med (Lausanne). 2020 Dec 18;7:581082. doi: 10.3389/fmed.2020.581082 (PMC7775734; doi:10.3389/fmed.2020.581082)
Supplement: Supplementary file 7 [file Image_2.pdf]

Supplementary Figure 2, Enrichment of KEGG pathways for differentially expressed genes in sepsis,

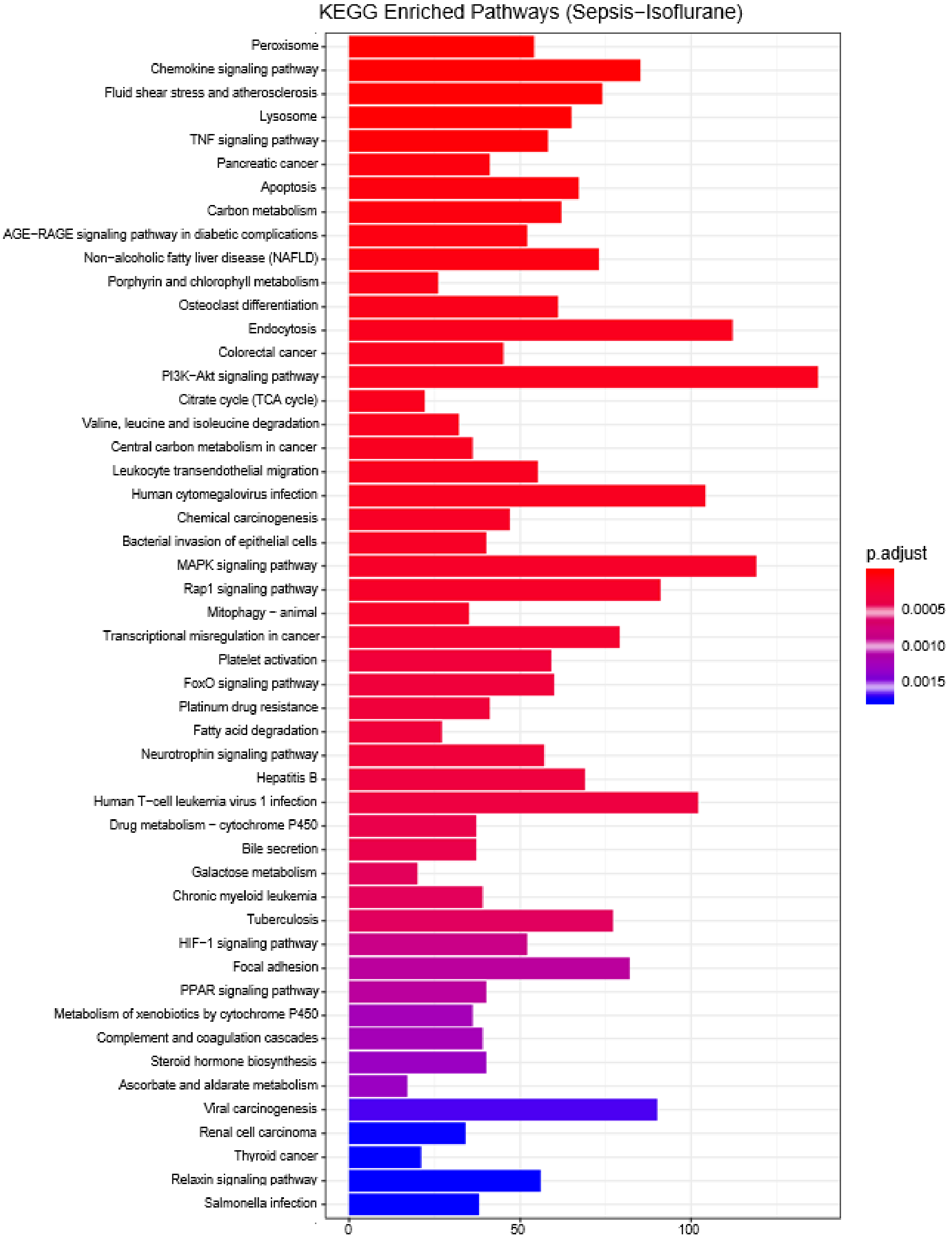

<u>Figure 2.</u> (A): Enrichment of KEGG pathways for differentially expressed genes in rat liver in response to Sepsis-inflammation with Isoflurane background: KEGG pathway enrichment p-value cutoff = 0.05; q-value cut off = 0.10; top 50; for differentially expressed genes the significance cut off level used was adjusted P-value > 0.05 for sepsis Vs. control. X-axis represents the gene counts. The most enriched pathways are those with the highest number of genes (e.g. PI3K-AKT signaling pathway in this figure).

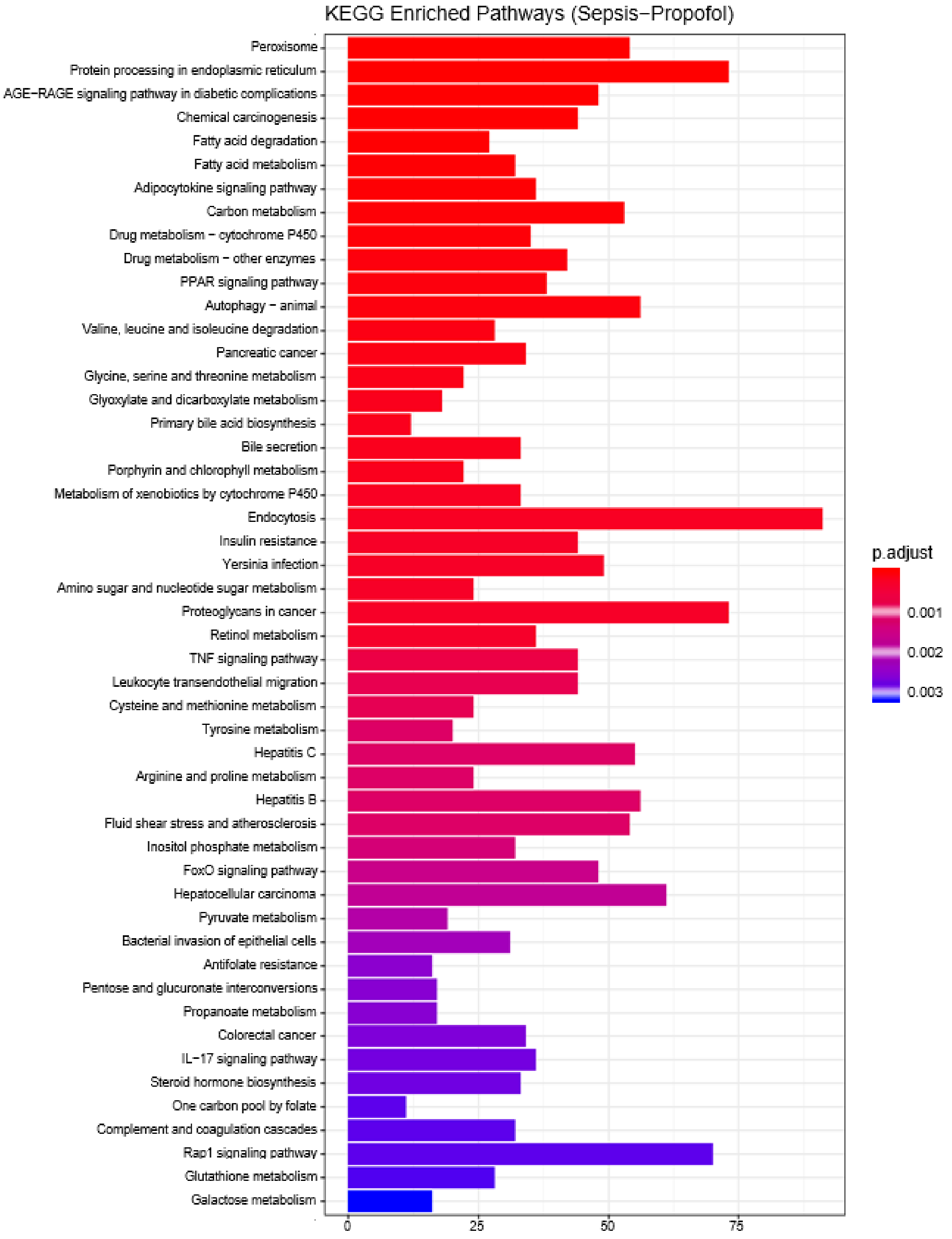

Figure 2. **(B): Enrichment of KEGG pathways for differentially expressed genes in rat liver in response to Sepsis-inflammation with Propofol background**: Significance cut off values are similar to S-Figure 5. A. The X-axis here represents the gene count.
